# Untargeted Metabolomics Reveals Intestinal Pathogenesis and Self-Repair in Rabbits Fed an Antibiotic-Free Diet

**DOI:** 10.3390/ani11061560

**Published:** 2021-05-27

**Authors:** Tao Tang, Ya Li, Jie Wang, Mauricio A. Elzo, Jiahao Shao, Yanhong Li, Siqi Xia, Huimei Fan, Xianbo Jia, Songjia Lai

**Affiliations:** 1 College of Animal Science and Technology, Sichuan Agricultural University, Chengdu 611130, China; m18483220592@163.com (T.T.); liya7911@163.com (Y.L.); wjie68@163.com (J.W.); shaojh1997@163.com (J.S.); lyh81236718@163.com (Y.L.); xiasiqi2020@163.com (S.X.); fanhuimei1998@163.com (H.F.); jaxb369@sicau.edu.cn (X.J.); 2 Department of Animal Sciences, University of Florida, Gainesville, FL 32611, USA; maelzo@ufl.edu; 3 Farm Animal Genetic Resources Exploration and Innovation Key Laboratory of Sichuan Province, Sichuan Agricultural University, Chengdu 611130, China

**Keywords:** antibiotic-free diet, intestinal inflammation, metabolomics, rabbit

## Abstract

**Simple Summary:**

In recent years, China imposed a total ban on the use of antibiotics in animal husbandry. This caused huge economic losses, one of the main reasons being an increase in the incidence of diseases. In this study, rabbits were used as a model to study the pathogenesis of intestinal diseases in rabbits on an antibiotic-free diet, through non-targeted metabolomics methods. The results showed that 1969 different metabolites were identified. These differential metabolites were involved in five metabolic pathways associated with intestinal inflammation (tryptophan metabolism, pyrimidine metabolism, phenylalanine, tyrosine and tryptophan biosynthesis, lysine degradation, and bile secretion). In summary, the use of non-antibiotic feed might cause intestinal inflammation in rabbits and activate intestinal repair.

**Abstract:**

The prohibition of the use of growth-promoting drug additives in feeds was implemented in China in 2020. However, rabbits can experience symptoms of intestinal disease, such as diarrhea and flatulence, when switching from standard normal diets with antibiotics to antibiotic-free diets. The molecular mechanisms related to the occurrence of these diseases as well as associated physiological and metabolic changes in the intestine are unclear. Thus, the objectives of this study were to study the pathogenesis of intestinal inflammation using untargeted metabolomics. This was done to identify differential metabolites between a group of antibiotic-free feed Hyplus rabbits (Dia) whose diet was abruptly changed from a standard normal diet with antibiotics to an antibiotic-free diet, and an antibiotic diet group Hyplus rabbits (Con) that was fed a standard normal diet with antibiotics. Morphological damage to the three intestinal tissues was determined through visual microscopic examination of intestinal Dia and Con tissue samples stained with hematoxylin and eosin (HE). A total of 1969 different metabolites were identified in the three intestinal tissues from Dia and Con rabbits. The level of 1280 metabolites was significantly higher and the level of 761 metabolites was significantly lower in the Dia than in the Con group. These differential metabolites were involved in five metabolic pathways associated with intestinal inflammation (tryptophan metabolism, pyrimidine metabolism, phenylalanine, tyrosine and tryptophan biosynthesis, lysine degradation, and bile secretion). Rabbits in the Dia group developed metabolic disorders that affected the intestinal microbiota and changed the permeability of the intestinal tract, thereby triggering intestinal inflammation, affecting feed utilization, reducing production performance, and activating the intestinal tract self-repair mechanism. Thus, the abrupt transition from a diet with antibiotics to an antibiotic-free diet affected the structure and metabolism of the intestinal tract in Hyplus rabbits. Consequently, to avoid these problems, the antibiotic content in a rabbit diet should be changed gradually or alternative antibiotics should be found.

## 1. Introduction

Clinical symptoms of intestinal disease in rabbits include diarrhea, dehydration, decreased appetite, and flatulence [1]. Causes of diarrhea in rabbits include bacteria [2,3], viruses [4], parasites [5], fungi [6], and environmental stress [7]. Abruptly changing a standard normal diet with antibiotics to an antibiotic-free diet is likely to alter the structure of the intestinal microbiota of young rabbits, and cause intestinal trauma and diarrhea, leading to death. In addition, the nontherapeutic usage of commercial antibiotics could cause tolerance or resistance in humans as well as animals [8]. Diarrhea and intestinal inflammation in rabbits are inseparable. Factors contributing to the stress caused by diarrhea in rabbits include noise, weaning, cold, heat, and pathogenic bacteria associated with enteritis and diarrhea present in the intestinal microbiota (e.g., clostridium difficile and clostridium perfringens) [9,10].

A large number of antibiotic growth promoters (AGPs) are regularly added to animal feed to meet productivity goals in intensive animal production systems [11]. However, the overuse of AGPs led to the development of antibiotic resistance in animal microbial populations and the possible transfer of antibiotic resistance genes from animals to the human microbiome [12]. Countries are increasingly banning the use of antibiotics in animal feeds [13]. China completely banned the use of antibiotics in animal feed in July 2020. However, the immaturity of antibiotic-free food technology plays an important role in the occurrence of intestinal diseases in livestock and poultry, particularly when switching from a standard normal diet to an antibiotic-free diet [14]. Intestinal diseases often manifest themselves as intestinal inflammation, thus the incidence of intestinal inflammation is frequently used as the primary base for judging the severity of gastrointestinal injuries [10,15,16,17]. Inflammation directly leads to impaired intestinal integrity [18]. Inflammation and damage to the intestine causes redistribution of nutrients, thereby, inhibiting the inflammatory response a fold-changed repair of the intestine. These events lead to a decline in animal productivity, and an increase in serious economic losses, due to intestinal diseases [19]. A few studies showed how the overall metabolites and metabolic pathways are related to intestinal inflammation in rabbits. High-throughput metabolomic techniques can be used to perform extremely sensitive experiments in a cost-effective manner [20]. Thus, the objectives of this study were to study the pathogenesis of intestinal inflammation, using untargeted metabolomics to identify differential metabolites in the intestine of Hyplus rabbits, after using antibiotic-free feed. Results from this study would provide a basis for the development of treatment of intestinal diseases in rabbits.

## 2. Materials and Methods

### 2.1. Farm Animal Sampling

Experimental procedures in this study were approved by the Institutional Animal Care and Use Committee of the College of Animal Science and Technology, Sichuan Agricultural University, China.

Two hundred Hyplus female rabbits at 35 days of age were chosen from the Zhongtian rabbit farm (Township, Leibo, Liangshan Yi Autonomous Prefecture, Sichuan, China) in three farm districts. Ten days before weaning, they were fed an antibiotic-containing feed together with female rabbits, and an antibiotic-free feed was freely fed for 10 days after weaning. All rabbits were raised in standard farm conditions and received a regular vaccine program. At the end of this experiment, 6 rabbits were screened out from normal state and treated as normal group (CON), while 6 rabbits were selected from sick rabbit under the selection of sick standard and treated as SICK group (DIA).

The selection of sick rabbit standard was according to phenotypic differences, stool and urine, weight differences, and intestinal histopathological diagnosis. Feed ingredients and additives was prepared according to the French INRA nutrient requirement, and its composition and nutrient content are shown in the supporting Table S1. Each rabbit was kept separately in a clean cage (600 × 600 × 500 mm) and placed in an environmentally controlled room (21–23 °C, 60–75% humidity, 14-h light [60 l×]).

### 2.2. Intestinal Tissue Sample Collection

Rabbits were euthanized by bloodletting with electroshock, after fasting for 24 h. Intestinal tissue samples for non-targeted metabolomics analysis were collected immediately after euthanasia. The contents of the intestine of each rabbit were washed with normal saline, prior to collecting colon, duodenum, and rectum intestinal tissue samples. Samples were placed into 3 mL cryotubes, and stored in liquid nitrogen at −80 °C.

### 2.3. Morphological Analysis of Intestinal Tissue

The intestinal tissue samples were dehydrated, embedded in paraffin, sectioned, and stained with hematoxylin and eosin (HE). The entire tissue slices were examined for histopathological changes under a microscope, and both normal areas and areas with obvious lesions were recorded with a microscopic imaging system.

### 2.4. Metabolite Extraction

Intestinal tissue samples (100 mg) were individually ground with liquid nitrogen and the homogenate was resuspended with prechilled 80% methanol and 0.1% formic acid using a well vortex. The samples were incubated on ice for 5 min and then were centrifuged at 15,000× *g* and 4 °C for 20 min. The supernatants were diluted with liquid chromatograph–mass spectrometer (LC–MS) grade water to a final concentration of 53% methanol. The samples were subsequently transferred to a fresh Eppendorf tube and were centrifuged at 15,000× *g* and 4 °C for 20 min. Lastly, the supernatants were injected into the ultra-high performance liquid chromatography–tandem mass-spectrometry (UHPLC-MS/MS) system for analysis [21].

### 2.5. UHPLC–MS/MS Analysis

The UHPLC–MS/MS analyses were performed using a Vanquish ultra-high pressure liquid chromatography (UHPLC) system (Thermo Fisher, Waltham, Germany) coupled with an Orbitrap Q Exactive ^TM^ HF mass spectrometer (Thermo Fisher, Germany) at Novogene Co., Ltd. (Beijing, China). Intestinal samples were injected into a Hypesil Gold column (100 × 2.1 mm, 1.9 μm), using a 17-min linear gradient with a flow rate of 0.2 mL/min. The eluents for the positive polarity mode were eluent A (0.1% formic acid in Water) and eluent B (Methanol). The eluents for the negative polarity mode were eluent A (5 mM ammonium acetate, pH 9.0) and eluent B (Methanol). The solvent gradient was set as follows—2% B, 1.5 m; 2 to 100% B, 12.0 m; 100% B, 14.0 m;100 to 2% B, 14.1 m; and 2% B, 17 m. The Orbitrap Q Exactive^TM^ HF mass spectrometer was operated in positive/negative polarity mode with a spray voltage of 3.2 kV, a capillary temperature of 320 °C, a sheath gas flow rate of 40 arb, and an aux gas flow rate of 10 arb.

### 2.6. Data Processing and Metabolite Identification

The raw data files generated by UHPLC-MS/MS were processed using Compound Discoverer 3.1 (CD3.1, Thermo Fisher) to perform peak alignment, peak picking, and quantitation for each metabolite. The main parameters were set as follows—retention time tolerance, 0.2 m; actual mass tolerance, 5 ppm; signal intensity tolerance, 30%; signal/noise ratio, 3; and minimum intensity, 100,000. Subsequently, peak intensities were normalized to total spectral intensity. The normalized data were used to predict molecular formulas, based on additive ions, molecular ion peaks, and fragment ions. Then, peaks were matched with the mzCloud (https://www.mzcloud.org/, accessed on 9 November 2020), mzVault, and MassList databases to obtain accurate qualitative and relative quantitative results. Statistical analyses were performed using the statistical software R (R version 3.4.3), Python (Python version 2.7.6), and CentOS (CentOS release 6.6). Chromatographic data were transformed using the area normalization method, when not normally distributed.

### 2.7. Metabolite Analysis

Metabolites were annotated with the KEGG (https://www.genome.jp/kegg/pathway.html/, accessed on 9 November 2020), The human metabolome database (HMDB) (https://hmdb.ca/metabolites/, accessed on 9 November 2020), and lipid metabolites and pathways strategy (LIPIDMaps) (http://www.lipidmaps.org/, accessed on 9 November 2020) databases. Principal components (PCA) and partial least squares discriminant (PLS–DA) analyses were performed using metaX, a flexible and comprehensive software for processing metabolomics data. Statistical significance (*p*-value) was determined using a univariate *t*-test. Metabolites with variable importance in projection (VIP) > 1, *p*-value < 0.05, and Fold Change > 1.5 or Fold Change < 0.667 were considered as differential metabolites. Volcano plots were used to identify the metabolites of interest, based on log_2_(Fold Change) and −log_10_(*p*-value). The data used for clustering the heat maps were normalized using z-scores of the intensity areas of differential metabolites, and plotted using the Pheatmap package in R. Correlations between differential metabolites were analyzed using the cor function in R (method = Pearson). Statistically significant correlations between differential metabolites were calculated with the cor.mtest R function. A *p*-value < 0.05 was considered to be statistically significant. Correlation plots were obtained with the corrplot package in R. The functions of differential metabolites and metabolic pathways were studied using the Kyoto encyclopedia of genes and genomes (KEGG) database. A metabolic pathway was considered to be enriched when (x/n) > (y/N) (observed metabolite frequency greater than KEGG expected metabolite frequency), and when *p* < 0.05 a metabolic pathway was considered to be significantly enriched. To further determine the biological significance of these differential metabolites, we used the MetaboAnalyst software (https://www.metaboanalyst.ca/, accessed on 9 November 2020) to conduct a metabolic pathway analysis.

## 3. Results

### 3.1. Intestinal Pathological Characteristics

**Figure 1:**
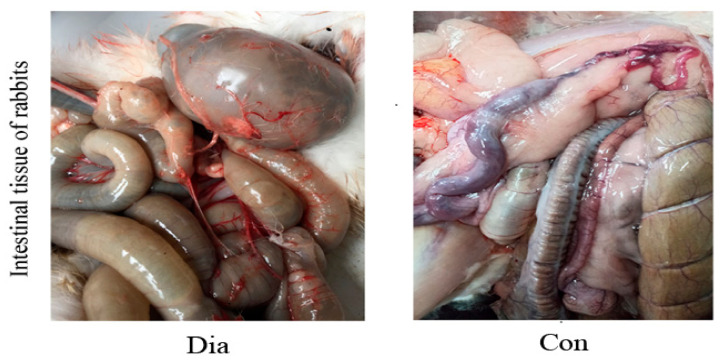
Intestinal anatomy of Hyplus rabbits fed either an antibiotic-free diet (Dia) or a standard normal diet (Con).

The anatomical structure of the intestine in the sick (Dia) and healthy (Con) Hyplus rabbit groups is shown in Figure 1. In addition, 6 rabbits in the Dia group showed the pathological features of diarrhea. The intestinal tract of the Dia group exhibited flatulence, the intestinal content had a substantial amount of water, and the intestinal wall was congested. The intestinal content of the Con group was dry, the anatomical structure was normal, the color of the intestinal wall was normal, and there was no hyperemia. These findings provide evidence for diarrhea, caused by intestinal pathology and intestinal microbial disorders in rabbits, due to a sudden change from a standard normal diet with antibiotics to an antibiotic-free diet.

**Figure 2:**
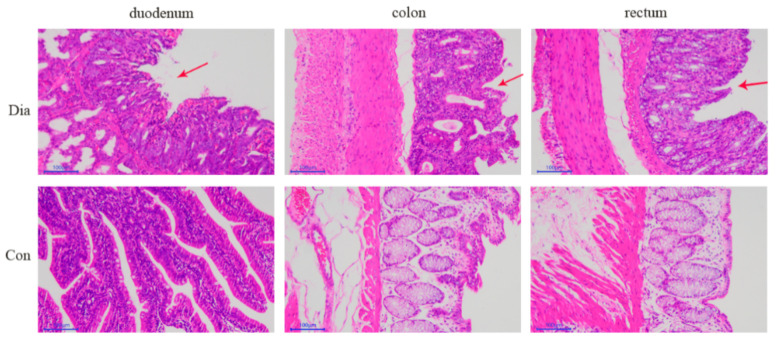
Duodenum, colon, and rectum intestinal tissue samples stained with hematoxylin and eosin (100×) from Hyplus rabbits fed either an antibiotic-free diet (Dia) or an antibiotic diet (Con). Red arrows point at the pathological features.

The HE-stained intestinal tissue samples (Figure 2) showed that the duodenal mucosa of rabbits in the Dia group was erosive with hemorrhage, the epithelial cells of the colon mucosa were locally necrotic and shedding, and the rectal mucosal epithelium was necrotic and shedding, forming erosions, and the lamina propria was slightly congested. Conversely, the intestinal structure of rabbits in the Con group was complete and without pathological features. These results indicated that a sudden change from a standard normal diet with antibiotics to one without antibiotics cause morphological damage to the intestinal tract in rabbits.

### 3.2. Screening and Acquisition of Primitive Metabolites

Characteristic molecular peaks in the intestinal tissue samples were detected using high-resolution mass spectrometry (HRMS) detection technology. We identified chromatographic peaks based on set mass deviations, signal-to-noise ratio, adduction, and quantification of peak areas. We then searched the mzCloud and mzVault high-resolution secondary spectrogram databases, and the MassList primary database to identify metabolites. Metabolites identified with a coefficient of variation (CV) lower than 30% in the quality control (QC) samples were retained for further analyses. We compared the retention time (RT), peak, intensity, and degree of separation from the total ion chromatogram (TIC) of the four QC cecum and the four colon samples in positive or negative ion modes. The TIC of the four QC samples overlapped well within each intestinal tissue, indicating that this method was robust, repeatable, and stable. The sample TIC showed complete peak shapes and good separation between the adjacent peaks, indicating that the chromatographic and mass spectrometry conditions were suitable for sample identification (Supplemental Figure S1). After screening, the number of metabolites that could be used for analysis were 1665 in the colon, 1791 in the duodenum, and 1748 in the rectum.

### 3.3. Quality Control of Metabolite Data from Intestinal Samples

The QC sample correlation analysis showed that the Pearson correlation coefficient [22] between the metabolite data from the four QC samples for each intestinal tissue was close to 1. The PCA of metabolite data from the Hyplus rabbits fed either an antibiotic-free diet (Dia group) or a standard normal diet (Con group) showed that the samples from the Dia and Con groups were well separated for the three intestinal tissues and that samples from the same rabbit group were clustered together (Supplementary Figure S2a). A Partial Least Squares Discriminant Analysis (PLS–DA) supervised model was used to assess intergroup sample differences. The PLS–DA score charts showed values of R^2^Y = 1.00 and Q^2^Y = 0.93 for colon, R^2^Y = 0.99 and Q^2^Y = 0.96 for duodenum, and R^2^Y = 1.00 and Q^2^Y = 0.94 for rectum tissue samples in the positive ion mode. The corresponding values of the PLS–DA score charts in the negative ion mode were R^2^Y = 1.00 and Q^2^Y = 0.91 for colon, R^2^Y = 0.99, and Q^2^Y = 0.95 for duodenum, and R^2^Y = 1.00 and Q^2^Y = 0.95 for the rectum tissue samples. Both R^2^Y and Q^2^Y values were approximately close to 1.0, indicating that the model was stable and reliable (Supplementary Figure S2b). The R^2^ data were greater than the Q^2^ data, and most Q^2^ values and the *Y*-axis intercept of the Q^2^ regression line were less than 0, indicating that the PLS–DA model did not overfit the data (Supplementary Figure S3b).

### 3.4. Differential Metabolite Analysis

**Figure 3:**
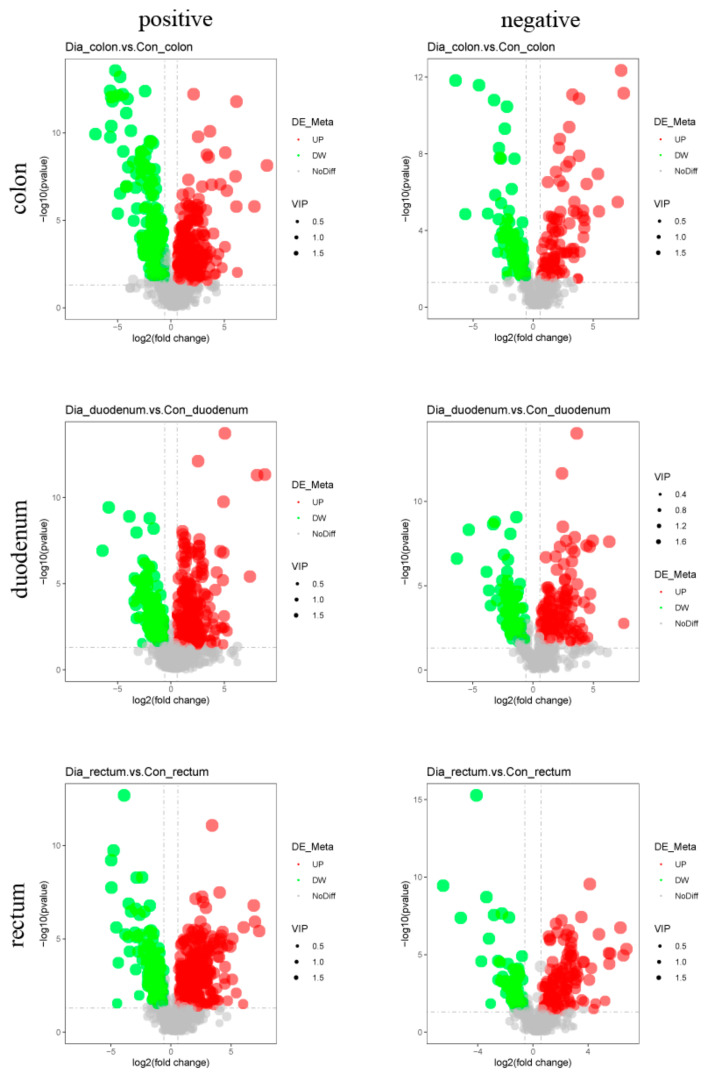
Volcano maps of differential metabolites in positive and negative ion modes from colon, duodenum, and rectum tissue samples from the Hyplus rabbits fed either an antibiotic-free diet (Dia group) or a standard normal diet (Con group). Volcano maps can visually display the overall distribution of different metabolites. Abscissas represent multiple differential metabolite changes between the Hyplus rabbit groups (log_2_ Fold Change). The ordinate represents the significance levels of differential metabolite changes between the Hyplus rabbit groups (−log_10_ *p*-value). Each point in the volcano plots represents a differential metabolite. The red dots represent significantly upregulated metabolites. The green dots represent significantly downregulated metabolites. Dot sizes represent variable importance in projection (VIP) values.

**Figure 4:**
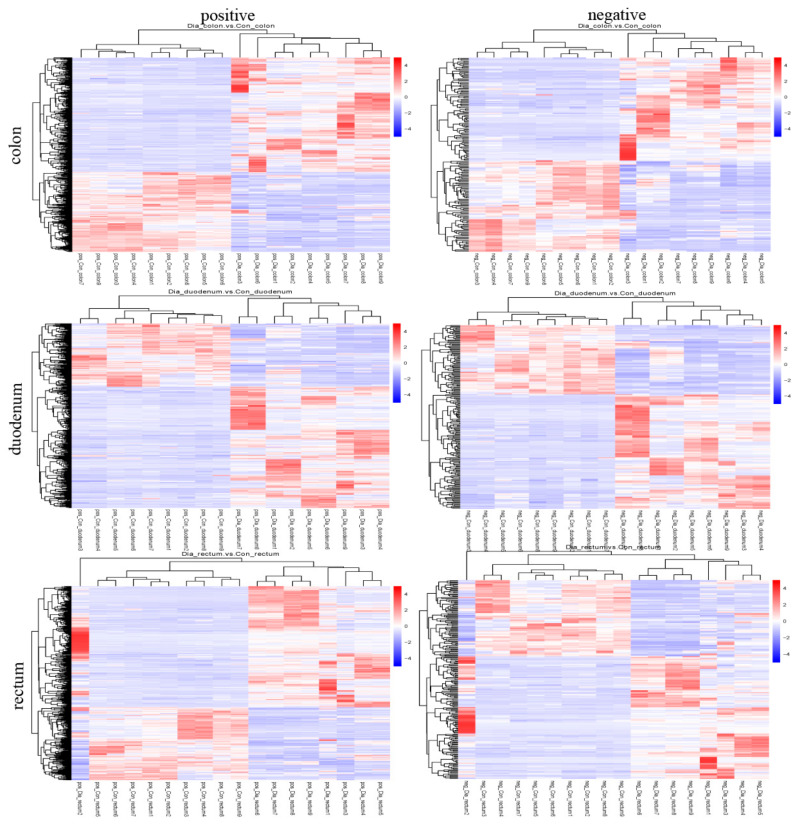
Cluster heat map of differential metabolites in positive and negative ion modes from colon, duodenum, and rectum tissue samples from the Hyplus rabbits fed either an antibiotic-free diet (Dia group) or a standard normal diet (Con group). Intestinal tissue samples are clustered vertically. Differential metabolites are clustered horizontally. The shorter the cluster branches, the higher the similarity. Links between horizontal clusters indicate relationships between differential metabolites from the Dia and Con rabbit groups.

The screening criteria used to identify differential metabolites in the colon, duodenum, and rectum of the Hyplus rabbits from the Dia and Con groups were VIP score > 1, Fold Change > 1.5, or Fold Change < 0.667, and *p*-value < 0.05. There were 651 differential metabolites in the colon (472 in the positive ion mode and 179 in the negative ion mode), 636 in the duodenum (432 in the positive ion mode and 204 in the negative ion mode), and 682 in the rectum (471 in the positive ion mode and 211 in the negative ion mode; Table 1). The content of 373 differential metabolites in the colon of rabbits from the Dia group was higher than that in the Con group, whereas the content of 278 differential metabolites was lower in the colon of Dia than Con rabbits. The content of 411 differential metabolites was higher and the content of 225 metabolites was lower in the duodenum of Dia than Con rabbits. Lastly, the content of 424 differential metabolites was higher and the content of 258 metabolites was lower in the rectum of Dia than Con rabbits (Figure 3). The cluster heat map showed that the distribution of differential metabolites in the Dia and Con groups was similar, and the dendrogram indicated that the colon, duodenum, and rectum tissue samples from rabbits in the Dia and Con groups could be separated (Figure 4), more detailed results are in Supplementary Figure S3.

### 3.5. KEGG Pathway Analysis

**Figure 5:**
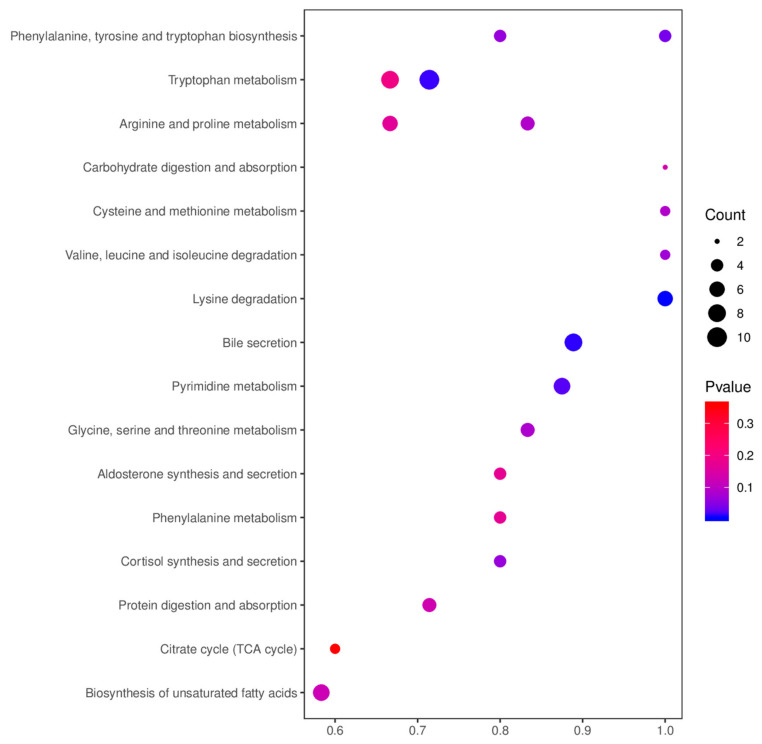
Bubble chart of the KEGG enrichment analysis of differential metabolites in positive and negative ion modes from colon, duodenum, and rectum tissue samples from the Hyplus rabbits fed either an antibiotic-free diet (Dia group) or a standard normal diet (Con group). The abscissa is the value of the ratio of the number of differential metabolites in a metabolic pathway divided by the total number of metabolites identified in the pathway. The larger the value, the higher the enrichment of differential metabolites in the pathway. The color of the dot represents the *p*-value of the hypergeometric test. The size of the dot represents the number of different metabolites in the pathway.

A total of 831 metabolites in the positive and negative ion modes were submitted to the KEGG pathway analysis, of which 332 were differential metabolites (Table 2). Key functional impacts of enriched differential metabolites on metabolic pathways were determined by the MetaboAnalyst (Figure 5). The enriched differential metabolites between the Dia and Con rabbit groups had a significant impact on five metabolic pathways, namely tryptophan metabolism, pyrimidine metabolism, phenylalanine, tyrosine and tryptophan biosynthesis, starch and sucrose metabolism, and bile secretion pathways (*p*-value < 0.05). In addition, the lysine degradation pathway in the Dia group was significantly different from that in the Con group (*p*-value < 0.01). There were ten metabolites involved in tryptophan metabolism, seven metabolites in pyrimidine metabolism, four metabolites in the biosynthesis of phenylalanine, tyrosine and tryptophan, three metabolites in the metabolism of starch and sucrose, eight metabolites involved in bile secretion, and six metabolites involved in the degradation of lysine.

## 4. Discussion

The promotion and use of antibiotic-free feed has become a trend in the development of new practices in animal husbandry. However, a complete switch from a standard normal diet with antibiotics to an antibiotic-free diet will cause severe diarrhea in rabbits, and the decline in production performance will bring great economic losses to the rabbit meat industry. This study explored factors influencing diarrhea caused by an abrupt change from a standard normal diet to an antibiotic-free diet in weaned Hyplus rabbits, using untargeted metabolomics [23,24]. The detected differential metabolites in the colon, duodenum, and rectum tissue samples of the Dia and Con Hyplus rabbit groups provided preliminary insights into the pathogenesis of diarrhea caused by a sudden switch from a standard normal diet with antibiotics to an antibiotic-free diet. Previous studies reported the occurrence of diarrhea associated with rapid diet changes in rabbit production systems [25]. One of the reasons for the high incidence of diarrhea in rabbits is related to insufficient secretion of endogenous enzymes. A second reason is that the intestinal environment of rabbits is complex, with a large number of microbes that can be easily stimulated and changed. Further, disorders of the intestinal microbiota are also an important factor leading to rabbit diarrhea [26,27]. Diarrhea leads to intestinal morphology damage and increases intestinal permeability. This allows a large numbers of microorganisms as well as toxic and harmful substances to invade the intestinal mucosa, causing intestinal inflammation [28,29]. The anatomical structure of the intestinal tract of Dia rabbits showed typical pathological characteristics of diarrhea. The amount of gas in the intestine was increased, fecal matter was washy, moisture increased, and the intestine was congested, indicating that the abrupt change of diet significantly affected their intestinal physiological metabolism.

Tryptophan metabolism was significantly different in the colon of Dia rabbits after switching to an antibiotic-free diet. Tryptophan is an essential amino acid in mammals that plays an important role in growth, development, and health of humans and animals. It is generally believed that tryptophan can regulate appetite in animals and promote growth and development [30]. In addition, there is also evidence that maintaining the stability of tryptophan metabolism can prevent and treat colorectal cancer (CRC) [31]. A study found that the content of indole and indole-3-acetic acid increased significantly in the tryptophan metabolic pathway [32]. Most gut Trp is converted to indole by tryptophan decarboxylase, to indole acetamide via tryptophan monooxygenase, and to tryptamine in the Trp indole pathway. Subsequently, these metabolites can be metabolized into skatole, IPA, or I3S, thus becoming end-products of the Trp metabolic pathway in the gut [33]. Tryptophan is involved in protein biosynthesis and is excreted through feces [32]. Tryptophan is metabolized into indole and indole derivatives by intestinal microorganisms, such as *Clostridium spores* and *E. coli* [34], *Achromobacter liquefaciens*, *Bacteroides* spp [35], and *Bifidobacterium* spp [36]. Jiang et al. [37] believe that suppression of the gut microbiome by addition of antibiotics in feed is likely the cause for the reduced level of phenylalanine metabolites, tryptophan metabolites from the serotonin pathway, and the secondary bile acids. These metabolites play an important role in regulating the expression of inflammation-related genes [38], enhancing the epithelial cell barrier [39], and inhibiting the growth of CRC cells in an aryl hydrocarbon receptor (AHR)-dependent manner [40]. These results are supported by several in vitro and in vivo studies [41]. Disruption of Trp indole AHR signaling led to significant increases in *TNF-α*, *IL-1β*, and *IL-6* mRNA levels in inflammatory colorectal tumor-generating models. Once activated by indoles, AHR acts directly on intestinal stem cells to enhance intestinal barrier function and maintain mucin production [42]. Exposure to physiological concentrations of indoles leads to increased expression of anti-inflammatory cytokines IL-10 [43] and IL-22 [44]. Therefore, indoles are involved in the intestinal barrier function. In addition, indoles have anticancer effects through the PI3K/Akt/mTOR signaling pathway [45]. Past studies showed that indole metabolites have an inhibitory effect on the occurrence of colorectal cancer. The use of antibiotic-free feeds increase the content of indole metabolites, activate the intestinal protection mechanism, and increase the intestinal barrier.

We found that the content of L-Hydroxylysine increased significantly, and hydroxylysine originates from degradation of proteins that have undergone post-translational modification from lysine to 5-hydroxylysine via lysyl hydroxylases (e.g., collagen) [46,47]. Then, free hydroxylysine is phosphorylated by HYKK (hydroxylysine kinase, also known as AGPHD1) and further converted to aminoadipate semialdehyde (AASA) [48]. Finally, it enters the metabolic pathway of lysine degradation. This indicates that the synthesis of lysine is reduced after feeding rabbits with an antibiotic-free diet. Lysine is an essential amino acid that participates in protein synthesis. Improving the efficient utilization of lysine for protein synthesis during animal growth can reduce feed costs and might reduce the input/output of nitrogen and phosphorus in animal production systems. Advanced biomolecular technology could be used to increase the efficiency of utilization of lysine, in order to synthesize proteins by reducing lysine degradation [49]. We can speculate that after a rabbit diet is abruptly changed from a diet with antibiotics to a diet without antibiotics, the intestinal tract becomes inflamed and the utilization rate of lysine decreases, which leads to a decrease in growth performance. However, the specific site of action, key enzymes involved, and impact mechanism need to be further investigated. However, it is worth noting that this article only provides a theoretical basis for the degradation of lysine, thus, the specific impact mechanism needs further exploration and verification.

Pyrimidine metabolism (Pym) involves a complex enzymatic hydrolysis network that integrates nucleoside recovery, de novo nucleotide synthesis, and pyrimidine catalytic degradation. Unlike dormant cells, cancer cells rely on a de novo approach to ensure a continuous supply of deoxyribonucleotide triphosphates (dNTPs) to support uncontrolled tumor growth [50,51]. Pym is a branch of nucleotide metabolism that produces nucleoside and pyrimidinyl (cytosine, thymine, and uracil) deoxyribonucleic acid. Along with purine metabolism, it produces a pool of deoxyribonucleotide needed for cell proliferation [52]. Carcinogenic effects of pyrimidine metabolites could be expected because purine molecules are proven to act as receptor ligands in the microenvironment of many tumors [53]. Extracellular purines, which generate signals through a set of specialized cell surface receptors, are classified as purine energy signals and might affect proliferation and metastasis [54,55]. We can also speculate and explore similar functions of pyrimidines. Endogenous accumulation of pyrimidine metabolites was reported in glycine decarboxylase-driven CD166^+^ cancer stem cells (CSCs) from non-small cell lung cancer (NSCLC) [56]. Therefore, the metabolites of pyrimidine metabolism are closely related to cancer. In this study, 5-methylcytosine (5 mC), a pyrimidine metabolite, was significantly different in the duodenum (*p*-value < 0.05). 5-methylcytosine (5 mC) was first discovered in CRC tumor tissue in 1988 [57]. Previous studies showed that low levels of 5 mC are closely related to advanced malignancy of breast and colon cancer [58,59]. However, the relationship between the expression of 5 mC and cancer did not yet appear in duodenum studies. In addition, early studies found that 5 mC is an independent marker of poor survival outcomes for CRC patients [60]. In this article, the expression of 5 mC was significantly upregulated (*p*-value < 0.05), which was different from previous report [61]. Possible reasons are differences in species and tissues. Thus, the relationship between changes in 5 mC metabolites and physiological effects in the duodenum needs further research.

The levels of amino acids (Anthranilic acid, L-Tryptophan, and L-Phenylalanine) were higher in Dia than in Con rabbits. Studies showed that under the treatment of antibiotics, the relative abundance and structure of *proteobacteria* in the mouse colon are changed, and the content of alanine and branched chain amino acids in the intestine is decreased [62]. Therefore, the use of antibiotics changed the link between gut microbes and amino acid metabolism. The intestinal microbiota has the ability to regulate the metabolic homeostasis of the host. Therefore, the use of antibiotics destroys the connection between the host and the microorganisms. There is evidence that most amino acids are synthesized and degraded in the liver [63], therefore, liver injuries can lead to abnormal amino acid metabolism and release of amino acids from liver cells [64]. Tryptophan and phenylalanine are aromatic amino acids that can be utilized by the intestinal microbiota to help decompose polyphenols and proteins in food [65]. Excessive release of tryptophan and phenylalanine disturbs the intestinal microbial metabolism and intestinal inflammation in rabbits [66].Hence, the reason for the increase in amino acids in Dia rabbits might be liver damage caused by a sudden change from a standard normal diet with antibiotics to an antibiotic-free diet. Interestingly, the content of pipecolic acid in the lysine degradation metabolic pathway was higher in the Dia than in the Con rabbit group. Furthermore, Fujita found that D-pipecolic and L-pipecolic acids were moderately elevated in patients with liver cirrhosis and chronic hepatic encephalopathy [67]. They also provided evidence for higher pipecolic acid levels in Dia rabbits, indicating that a sudden change from an antibiotic-containing diet to another non-antibiotic-free diet might have caused damage to the liver of these rabbits. However, the specific action mechanisms involved need to be further verified.

Bile acids (BA) are the general term for a large class of cholanic acids, which exist in the form of sodium or potassium salts. They are amphipathic cholesterol metabolites synthesized in the liver, stored in the gallbladder, and then released in the intestinal tract. Bile acids have many important physiological functions. Bile acid oligomers and their combination with antibiotics are used to combat bacterial infections [68]. Early studies found that bile acids can be used as an emulsion to promote the absorption of fatty acids and fat-soluble vitamins [69]. In addition, the intestinal microbiota in humans and animals is essential for the conversion of bile acids. The bile acids secreted by hepatocytes are “primary” BA. After they enter the intestine, bacteria in the ileum and upper colon convert the primary BA into “secondary” BA such as deoxycholic acid (DA), lithocholic acid (LCA), and ursodesoxycholic acid (UDCA), through a sequence of processes including oxidation, reduction, hydroxylation, and dihydroxylation [70]. The elucidation of the regulatory pathways of BA circulation between the intestine and liver (enterohepatic circulation) and the identification of BA-specific receptors in a variety of cell types and tissues emphasized their role in health [71]. The pathogenesis of inflammatory bowel disease (IBD) is closely related to an increased permeability of intestinal epithelial cells to luminal macromolecules [72]. In addition, BAs are likely to play a role in the enhanced epithelial permeability that is associated with the progression of intestinal diseases [73]. Thus, changes in BA levels are likely related to the pathogenesis of the IBD [74]. The level of LCA was significantly reduced in the bile secretion metabolic pathway of the Dia rabbit group, as compared to that in the Con group in this study (*p*-value < 0.05). Previous research using the dextran sodium sulfate (DSS) model of intestinal disease showed that the initiation of intestinal inflammation was due to a breakdown of the epithelial barrier function [75]. Conversely, LCA and its tauro ursodesoxy cholic acid (TUDCA) were found to have a protective effect on the intestinal epithelial barrier in preclinical models of intestinal inflammation [76]. Thus, this study proved the relationship between intestinal inflammation and BAs in rabbits for the first time. However, the specific mechanism of bile secretion and intestinal inflammation needs further study.

**Figure 6:**
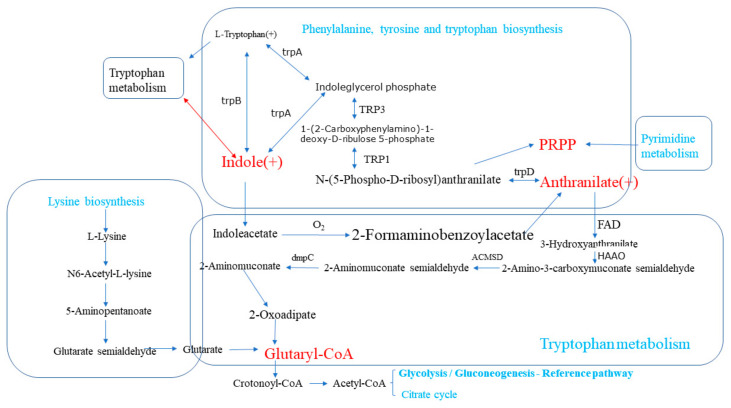
Relationships among differential metabolic pathways associated with intestinal inflammation in Hyplus rabbits fed either an antibiotic-free diet (Dia group) or a standard normal diet (Con group). The boxes represent different metabolic pathways. Substances in red represent key metabolites between metabolic pathways. (+) indicates an increase in the content of a substance. Substances in green represent the names of the metabolic pathways.

The most important differential metabolic pathways associated with intestinal inflammation found in this study were tryptophan metabolism, phenylalanine, tyrosine, and tryptophan biosynthesis. These two metabolic pathways share a common metabolite—indole—an upregulated metabolite in the Dia rabbit group that has become one of the hubs of the metabolic pathways involved in intestinal inflammation (Figure 6). Therefore, after changing the diet to an antibiotic-free feed, it has an impact on the various metabolic pathways of rabbits, induces intestinal inflammation, and activates the intestinal self-repair mechanism.

## 5. Conclusions

A sudden change from a standard normal diet with antibiotics to an antibiotic-free diet can cause major changes in rabbit intestinal metabolism. First, it can activate the protective mechanism of the intestine, increase the content of indole, repair intestinal damage, and increase the effectiveness of the intestinal barrier. Second, it alters the bile metabolism in the liver by reducing the LCA levels and increasing the L-tryptophan levels. These changes disrupt the metabolism of the intestinal microbiota, leading to intestinal inflammation. Third, it reduces the utilization of lysine in the feed and hinders the synthesis of protein, thereby affecting rabbit growth performance. The results of this study provide a theoretical basis for the pathogenesis of rabbit intestinal disease at the metabolome level, and lay the foundation for the development of drugs for the treatment of livestock diarrhea.

## Figures and Tables

**Table 1:** Number of differential metabolites in colon, duodenum, and rectum intestinal samples from the Hyplus rabbits fed either an antibiotic-free diet (Dia group) or a standard normal diet (Con group).

Intestinal Tissue Comparison	Ion Mode	N Total Identified ^1^	N Signif Different ^2^	N Signif Up ^3^	N Signif Down ^4^
Dia_colon vs. Con_colon	Positive	1191	472	278	194
	Negative	472	179	95	84
	Sum	1663	651	373	278
Dia_duodenum vs. Con_duodenum	Positive	1096	432	284	148
	Negative	493	204	127	77
	Sum	1589	636	411	225
Dia_rectum vs. Con_rectum	Positive	1224	471	294	177
	Negative	522	211	130	81
	Sum	1746	682	424	258
All intestinal tissue comparisons	Total	4998	1969	1208	761

^1^ N Total Identified = Total number of differential metabolites between Dia and Con rabbits. ^2^ N Signif Different = Number of significant differential metabolites between Dia and Con rabbits. ^3^ N Signif Up = Number of significantly higher metabolite contents in Dia than in Con rabbits. ^4^ N Signif Down = Number of significantly lower metabolite contents in Dia than in Con rabbits.

**Table 2:** KEGG enrichment analysis of differential metabolites from colon, duodenum, and rectum intestinal tissue samples from the Hyplus rabbits fed either an antibiotic-free diet (Dia group) or a standard normal diet (Con group) ^1^.

Tissue	Map ID	Map Title	*p*-Value	N	Meta IDs
colon	map04976	Bile secretion	0.204218	94	Salicylic acid, Deoxycholic acid, Lithocholic Acid, and Chenodeoxycholic Acid.
	map00400	Phenylalanine, tyrosine and tryptophan biosynthesis	0.063043	184	Anthranilic acid, Phenylpyruvic acid, L-Tryptophan, and Indole.
	map04927	Cortisol synthesis and secretion	0.063043	184	Cortisol, Pregnenolone, Progesterone, and Cortodoxone.
	map04934	Cushing’s syndrome	0.063043	184	Cortisol, Pregnenolone, Progesterone, and Cortodoxone.
duodenum	map00330	Arginine and proline metabolism	0.089757	159	Creatine, Spermine, S-Adenosylmethionine, Creatinine, and L-Glutamic acid.
	map00360	Phenylalanine metabolism	0.173262	159	2-Phenylacetamide, Phenylacetylglycine, L-Phenylalanine, and D-Phenylalanine.
	map00380	Tryptophan metabolism	0.195911	159	Anthranilic acid, L-Tryptophan, N-Acetylserotonin, Kynurenic acid, Xanthurenic acid, 5-Hydroxyindoleacetate, N-Formylkynurenine, and Indole.
duodenum	map00053	Ascorbate and aldarate metabolism	0.197835	159	L-Ascorbate and UDP-D-glucuronate.
	map00270	Cysteine and methionine metabolism	0.089654	106	L-Aspartic acid, Glutathione, and Reduced Glutathione.
	map04925	Aldosterone synthesis and secretion	0.173475	106	cGMP, Aldosterone, Pregnenolone, and NAD+.
rectum	map00220	Arginine biosynthesis	0.070142	178	L-Glutamic acid, N-Acetyl-L-glutamic acid, and L-Ornithine.
	map00280	Valine, leucine and isoleucine degradation	0.070142	178	3-Methyl-2-oxobutanoic acid, TPP, and Acetoacetate.
	map00260	Glycine, serine and threonine metabolism	0.083262	178	O-Phospho-L-serine, Creatine, Betaine, L-Tryptophan, and L-Cystathionine.
	map04974	Protein digestion and absorption	0.128992	178	Indole, L-Asparagine, Histamine, L-Tryptophan, and Tyramine.
	map00330	Arginine and proline metabolism	0.166117	178	L-Glutamic acid, Spermine, Creatine, Creatinine, N-Methylhydantoin, and L-Ornithine.
	map01040	Biosynthesis of unsaturated fatty acids	0.125115	110	Nervonic acid, Docosanoic acid, Adrenic acid, Stearic acid, Erucic acid, Arachidic acid, and Docosahexaenoic acid.
	map04973	Carbohydrate digestion and absorption	0.136781	110	Maltotriose and D-Glucose 6-phosphate.
	map00020	Citrate cycle (TCA cycle)	0.35937	110	Cis-Aconitic acid, L-Malate, and Fumaric acid.

^1^ Map ID = ID of enriched KEGG pathway; Map Title = Name of enriched KEGG pathway; and N = Number of backg.

## Data Availability

All data generated or analyzed during this study are included.

## Supplementary Materials

The following are available online at https://www.mdpi.com/article/10.3390/ani11061560/s1. Figure S1: Total ion chromatogram of plasma samples analyzed in the positive and negative ion modes. Figure S2a: Principal component analysis of metabolite data from colon, duodenum, and rectum tissue samples from the Hyplus rabbits fed either an antibiotic-free diet (Dia group) or a standard normal diet (Con group). Positive ion mode on the left and negative ion mode on the right. The x-coordinate PC1 and y-coordinate PC2 represent the scores of the first and second principal components. The red scatter points represent intersections of PC1 and PC2 for intestinal tissue samples from Con rabbits and the green scatter points represent intersections of PC1 and PC2 for intestinal tissue samples from Dia rabbits. Ellipses are 95% confidence intervals. Figure S2b: Scatter point and sort verification plots of PLS–DA score data for colon, duodenum, and rectum tissue samples from the Hyplus rabbits fed either an antibiotic-free diet (Dia group) or a standard normal diet (Con group). (A) PLS–DA scatter plots. Abscissas contain PLS–DA scores for the first principal component of intestinal tissue samples. Ordinates contains PLS–DA scores for the second principal component of intestinal tissue samples. R2Y represents the interpretation rate of the PLS–DA model. Q2Y assesses the predictive ability of the PLS–DA model. Higher R2Y than Q2Y values indicate that the PLS–DA model is stable. (B) PLS–DA sort verification plots. Abscissas contain correlations between random group and original group Y values. Ordinates contain PLS–DA R2 and Q2 scores. Figure S3: Detailed graphs of clustering heat maps of differential metabolite positive and negative ion patterns in Hyplus rabbit colon, duodenum, and rectal tissue samples in the antibiotic-free diet group (Dia group) and the standard normal diet group (Con group., Table S1: Feed formula and main nutritional indicators of standard diet group (Con) contains antibiotics and no antibiotic group (Dia).
