# Supplementary material for: Untargeted Metabolomics Reveals Intestinal Pathogenesis and Self-Repair in Rabbits Fed an Antibiotic-Free Diet
Source: Animals (Basel). 2021 May 27;11(6):1560. doi: 10.3390/ani11061560 (PMC8228699; doi:10.3390/ani11061560)
Supplement: Supplementary file 1 [file animals-11-01560-s001.zip › animals-1196821-supplementary-update/animals-1147480-supplementary/Supplemental Figure 1 Total ion chromatogram of plasma samples analyzed in the positive and negative ion modes/Figure S1.pdf]

Cecum posQC

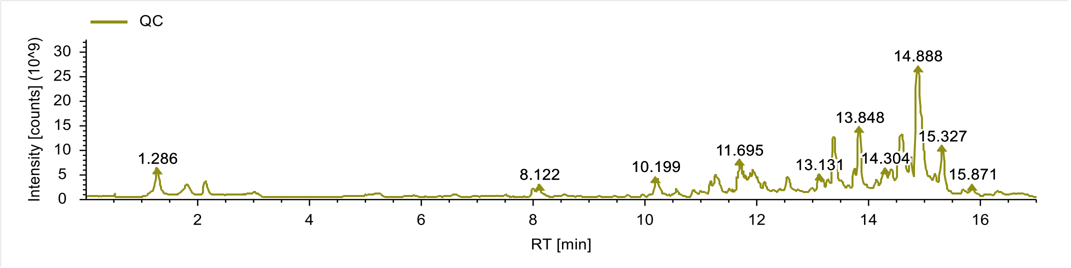

Cecum necQC

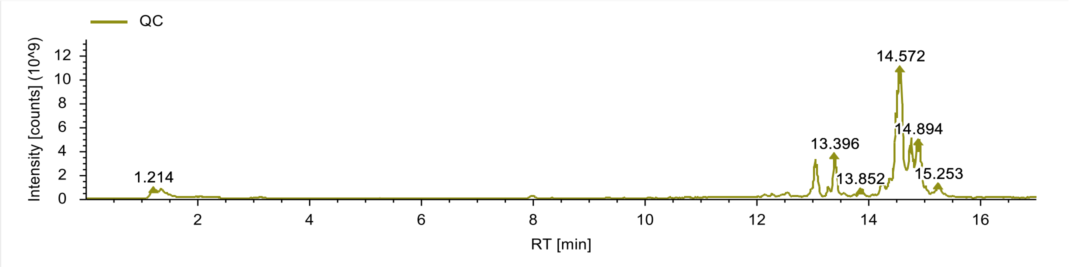

Colon posQC

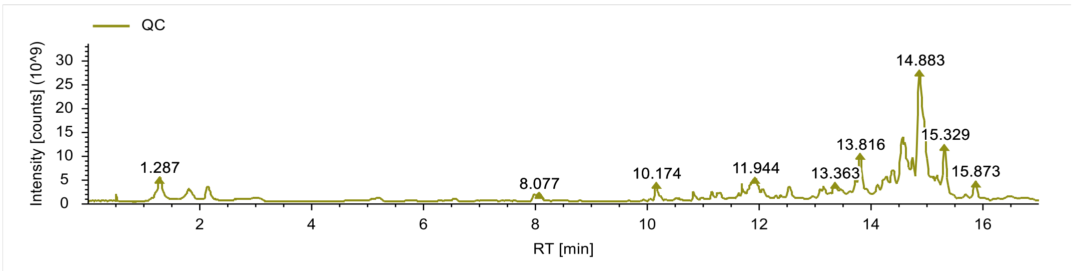

colon negQC

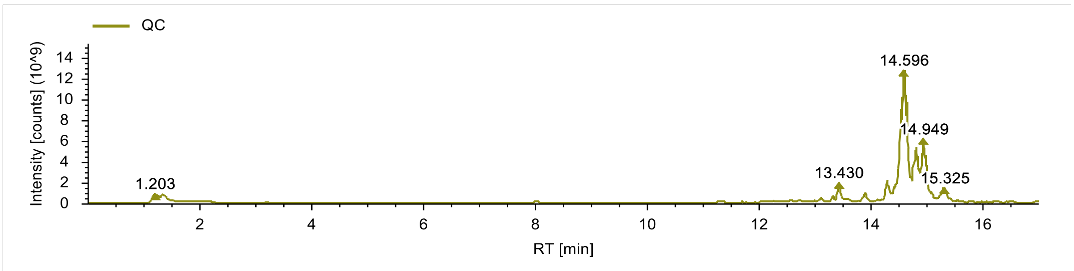

Supplemental Figure S1. Total ion chromatogram of plasma samples analyzed in the positive and negative ion modes.
