# Supplementary material for: Untargeted Metabolomics Reveals Intestinal Pathogenesis and Self-Repair in Rabbits Fed an Antibiotic-Free Diet
Source: Animals (Basel). 2021 May 27;11(6):1560. doi: 10.3390/ani11061560 (PMC8228699; doi:10.3390/ani11061560)
Supplement: Supplementary file 1 [file animals-11-01560-s001.zip › animals-1196821-supplementary-update/animals-1147480-supplementary/Supplementary table 1.pdf]

Supplementary table 1 The pathological characteristics of colon, duodenum, rectum and intestinal tract of Dia and Con were obtained by HE staining.

Group	Pathological feature description		
	colon	duodenum	rectum
Dia-1[1]	Slight congestion of mucosal capillaries	Full-thickness intestinal wall necrosis	Increase in lymphocytes in the submucosa
Dia-2	Destruction of tissue structure, full-thickness coagulation necrosis, tissue contours are still visible in the mucosal layer, submucosa, muscle layer and serosal layer, and large parts of the necrotic cell nuclear fragments		
Dia-3	Local necrosis and shedding of the mucosal epithelium formed erosions, and the lamina propria was slightly hyperemic	Mucosal erosion with bleeding.	The lamina propria is slightly congested
Dia-4	The structure of the mucosa is complete, some epithelial cells are swollen, and the nucleus is in the center	Mucosal layer necrosis, crypts and intestinal gland tissue structure destruction	The tissue structure is relatively complete without obvious pathological damage
Dia-5	Local necrosis and shedding of epithelial cells in the mucosal layer	The tissue structure is relatively normal, and there is no obvious pathological damage	
Dia-6	The tissue structure is relatively normal, and there is no obvious pathological damage	Full-thickness intestinal wall necrosis	The tissue structure is relatively normal, and there is no obvious pathological damage
Con 1-6	The structure of the tissue sample is complete, the cells are arranged neatly, and there is no obvious histopathological damage		

1 There are a total of 12 rabbits in the Dia group and Con group, and the pathological characteristics of each rabbit's intestinal tract are described separately.
