# Supplementary material for: Untargeted Metabolomics Reveals Intestinal Pathogenesis and Self-Repair in Rabbits Fed an Antibiotic-Free Diet
Source: Animals (Basel). 2021 May 27;11(6):1560. doi: 10.3390/ani11061560 (PMC8228699; doi:10.3390/ani11061560)
Supplement: Supplementary file 1 [file animals-11-01560-s001.zip › animals-1196821-supplementary-update/animals-1147480-supplementary/Supplementary table 2.pdf]

Supplementary table 2 Feed formula and main nutritional indicators of standard diet group (Con) contains antibiotics and no antibiotic group (Dia).

<table>
<tr><th rowspan="2">Ingredient</th><th>Con</th><th>Dia</th><th rowspan="2">Nutrient content</th><th>Con</th><th>Dia</th></tr>
<tr><th colspan="2">Proportion (%)</th><th colspan="2">Content</th></tr>
<tr><td>Bean cake</td><td colspan="2">76.9</td><td>Digestible energy</td><td colspan="2">13.5KJ/Kg</td></tr>
<tr><td>Fish meal</td><td colspan="2">19.9</td><td>Crude protein</td><td colspan="2">45.0%</td></tr>
<tr><td>Salt</td><td colspan="2">2</td><td>Lysine</td><td colspan="2">2.9%</td></tr>
<tr><td>Trace elements</td><td colspan="2">0.2</td><td>Cystine and Methionine</td><td colspan="2">1.6%</td></tr>
<tr><td>Vitamins</td><td colspan="2">0.1</td><td>Salt</td><td colspan="2">2%</td></tr>
<tr><td>Lysine</td><td colspan="2">0.5</td><td></td><td colspan="2"></td></tr>
<tr><td>Methionine</td><td colspan="2">0.4</td><td></td><td colspan="2"></td></tr>
<tr><td>Robenidine Hydrochloride</td><td>0.015</td><td></td><td></td><td colspan="2"></td></tr>
<tr><td>Sulfaguanidine</td><td>0.07</td><td></td><td></td><td colspan="2"></td></tr>
</table>
