# Supplementary material for: Untargeted Metabolomics Reveals Intestinal Pathogenesis and Self-Repair in Rabbits Fed an Antibiotic-Free Diet
Source: Animals (Basel). 2021 May 27;11(6):1560. doi: 10.3390/ani11061560 (PMC8228699; doi:10.3390/ani11061560)
Supplement: Supplementary file 1 [file animals-11-01560-s001.zip › animals-1196821-supplementary-update/animals-1147480-supplementary/Supplementary table 3.pdf]

Supplementary table 3 A list of abbreviations would be to define all abbreviations used in the study.

Abbreviations	Full names
Dia	antibiotic-free feed group
Con	antibiotic diet group
HE	haematoxylin and eosin
UHPLC-MS/MS	ultra-high performance liquid chromatographytandem mass-spectrometry
LC-MS	liquid chromatograph-mass spectrometer
UHPLC	ultra-high pressure liquid chromatography
HMDB	the human metabolome database
LIPIDMaps	lipid metabolites and pathways strategy
PCA	Principal components analysis
PLS-DA	partial least squares discriminant analysis
VIP	variable importance in projection
KEGG	kyoto encyclopedia of genes and genomes
HRMS	high resolution mass spectrometry
CV	coefficient of variation
QC	quality control
TIC	total ion chromatogram
RT	retention time
CRC	colorectal cancer
AHR	aryl hydrocarbon receptor
AASA	aminoadipate semialdehyde
HYKK	hydroxylysine kinase
Pym	Pyrimidine metabolism
dNTPs	deoxyribonucleotide triphosphates
CSCs	cancer stem cells
5mC	5-methylcytosine
DSS	dextran sodium sulfate
BA	bile acids
DA	deoxycholic acid
LCA	lithocholic acid
UDCA	ursodesoxycholic acid
IBD	inflammatory bowel disease
TUDCA	tauro ursodesoxy cholic acid
