# Supplementary figures and images for: Untargeted Metabolomics Reveals Intestinal Pathogenesis and Self-Repair in Rabbits Fed an Antibiotic-Free Diet
Source: Animals (Basel). 2021 May 27;11(6):1560. doi: 10.3390/ani11061560 (PMC8228699; doi:10.3390/ani11061560)

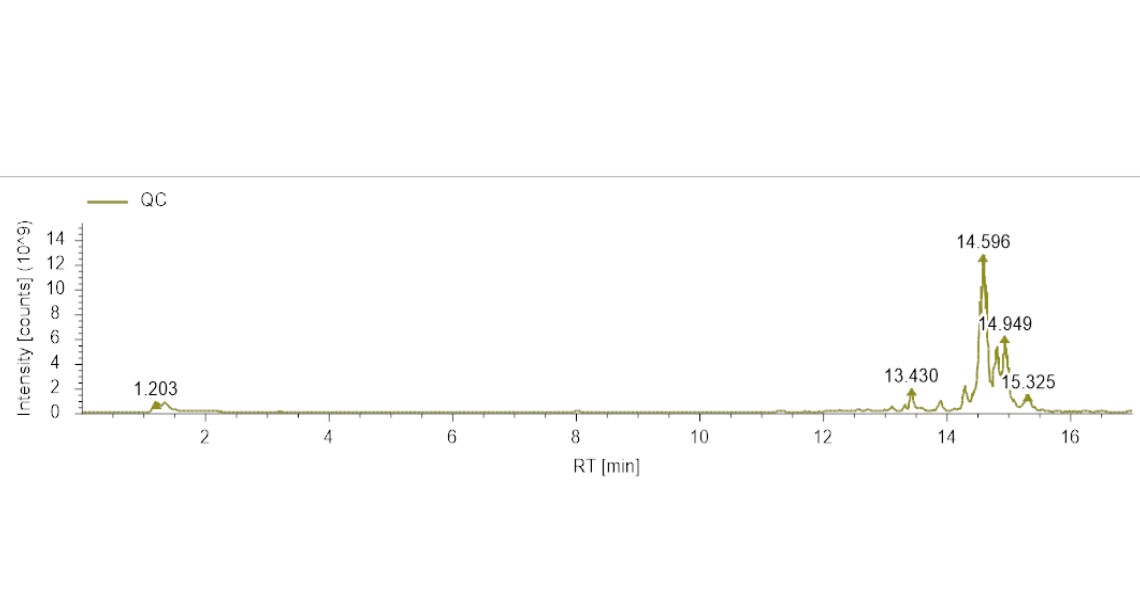

Supplement: Supplementary file 1 [file animals-11-01560-s001.zip › animals-1196821-supplementary-update/animals-1147480-supplementary/Supplemental Figure 1 Total ion chromatogram of plasma samples analyzed in the positive and negative ion modes/A-colon-neg.jpg]

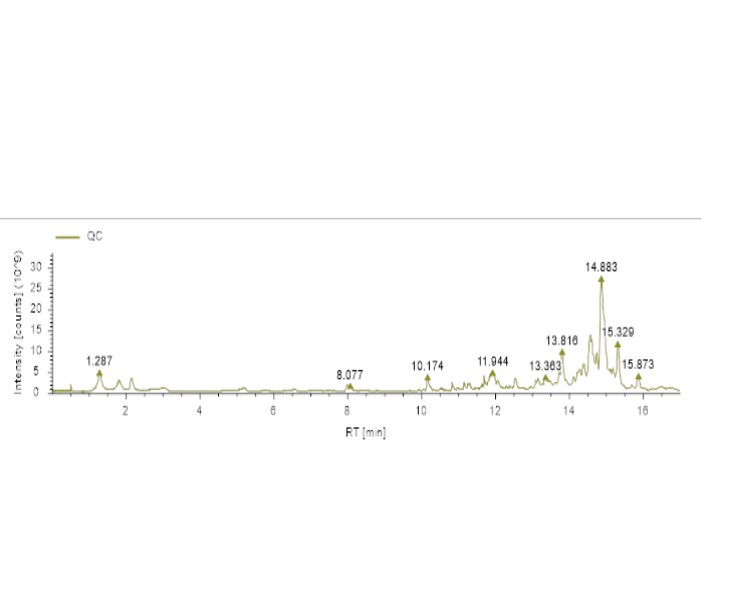

Supplement: Supplementary file 1 [file animals-11-01560-s001.zip › animals-1196821-supplementary-update/animals-1147480-supplementary/Supplemental Figure 1 Total ion chromatogram of plasma samples analyzed in the positive and negative ion modes/A-colon-pos.jpg]

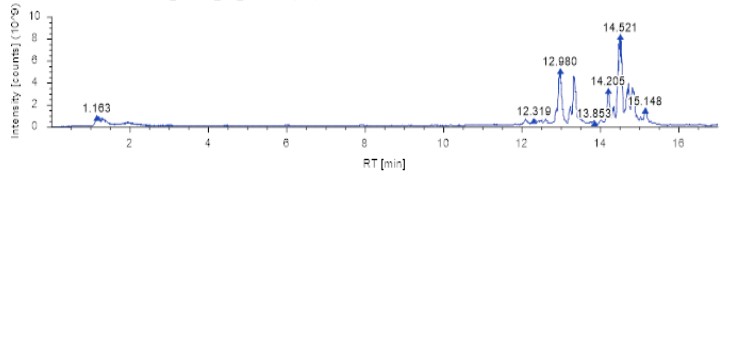

Supplement: Supplementary file 1 [file animals-11-01560-s001.zip › animals-1196821-supplementary-update/animals-1147480-supplementary/Supplemental Figure 1 Total ion chromatogram of plasma samples analyzed in the positive and negative ion modes/B-duodenum-neg.jpg]

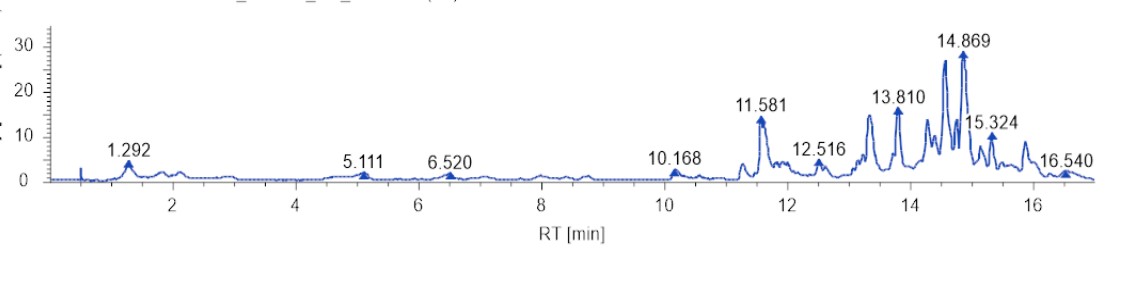

Supplement: Supplementary file 1 [file animals-11-01560-s001.zip › animals-1196821-supplementary-update/animals-1147480-supplementary/Supplemental Figure 1 Total ion chromatogram of plasma samples analyzed in the positive and negative ion modes/B-duodenum-pos.jpg]

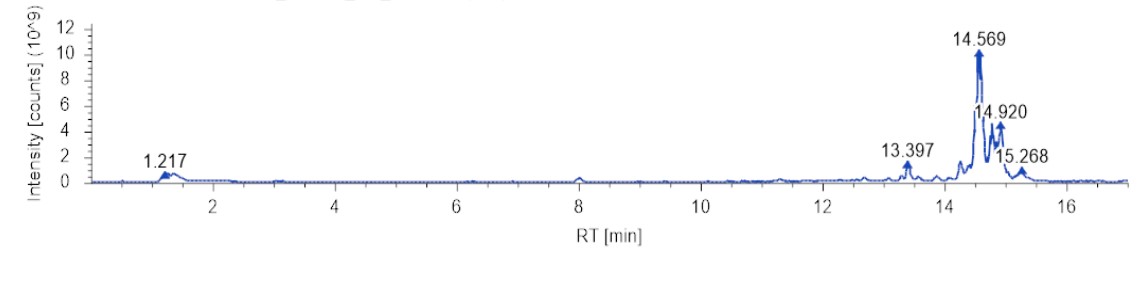

Supplement: Supplementary file 1 [file animals-11-01560-s001.zip › animals-1196821-supplementary-update/animals-1147480-supplementary/Supplemental Figure 1 Total ion chromatogram of plasma samples analyzed in the positive and negative ion modes/C-rectum-neg.jpg]

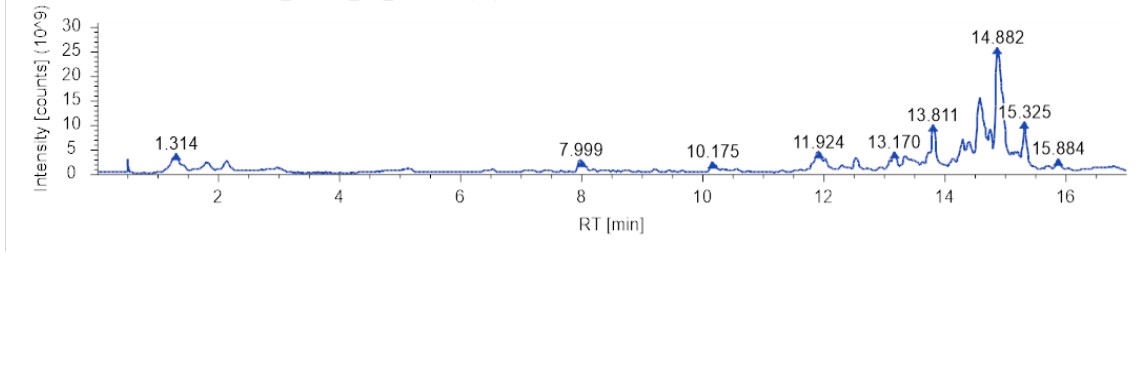

Supplement: Supplementary file 1 [file animals-11-01560-s001.zip › animals-1196821-supplementary-update/animals-1147480-supplementary/Supplemental Figure 1 Total ion chromatogram of plasma samples analyzed in the positive and negative ion modes/C-rectum-pos.jpg]

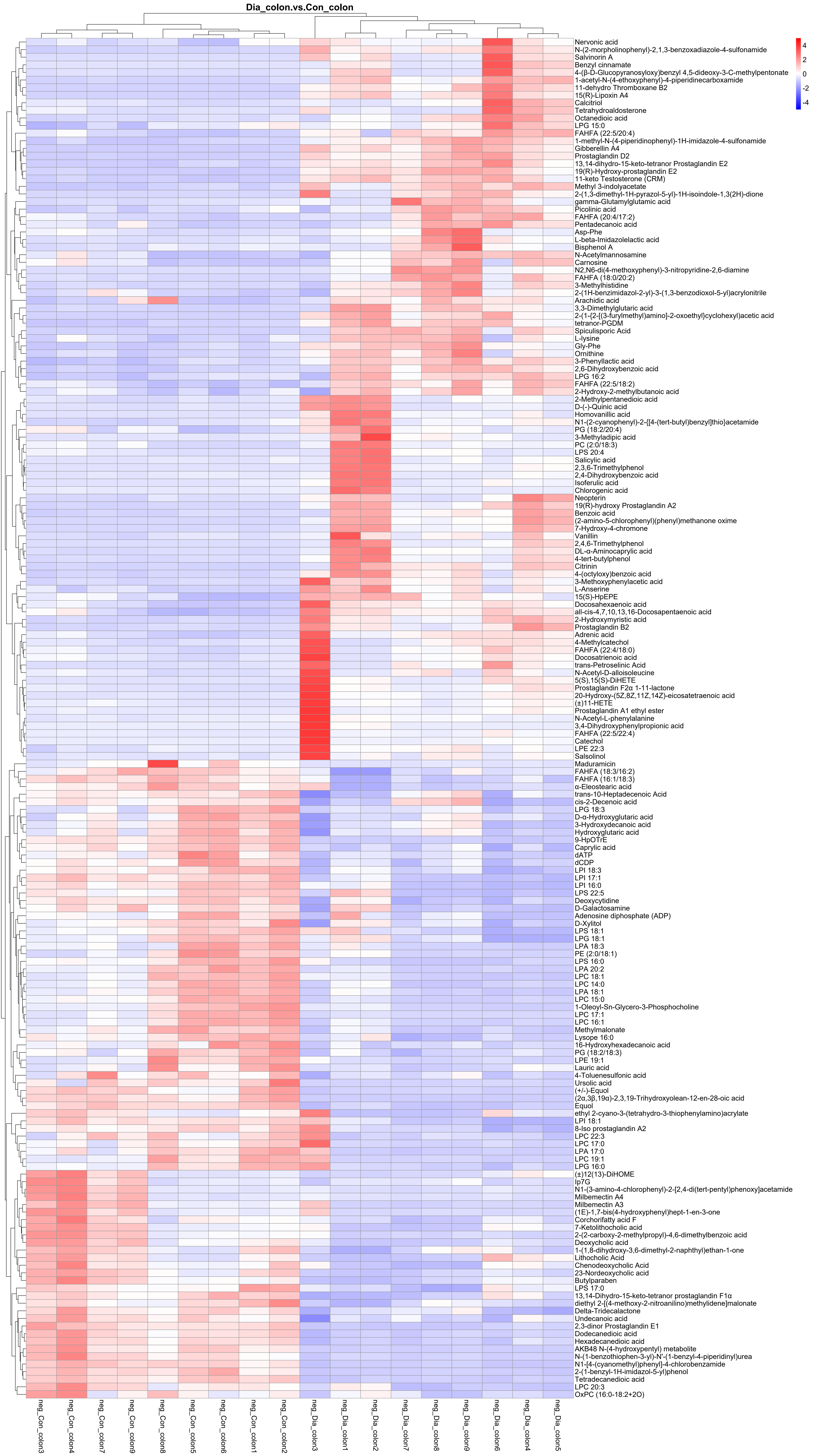

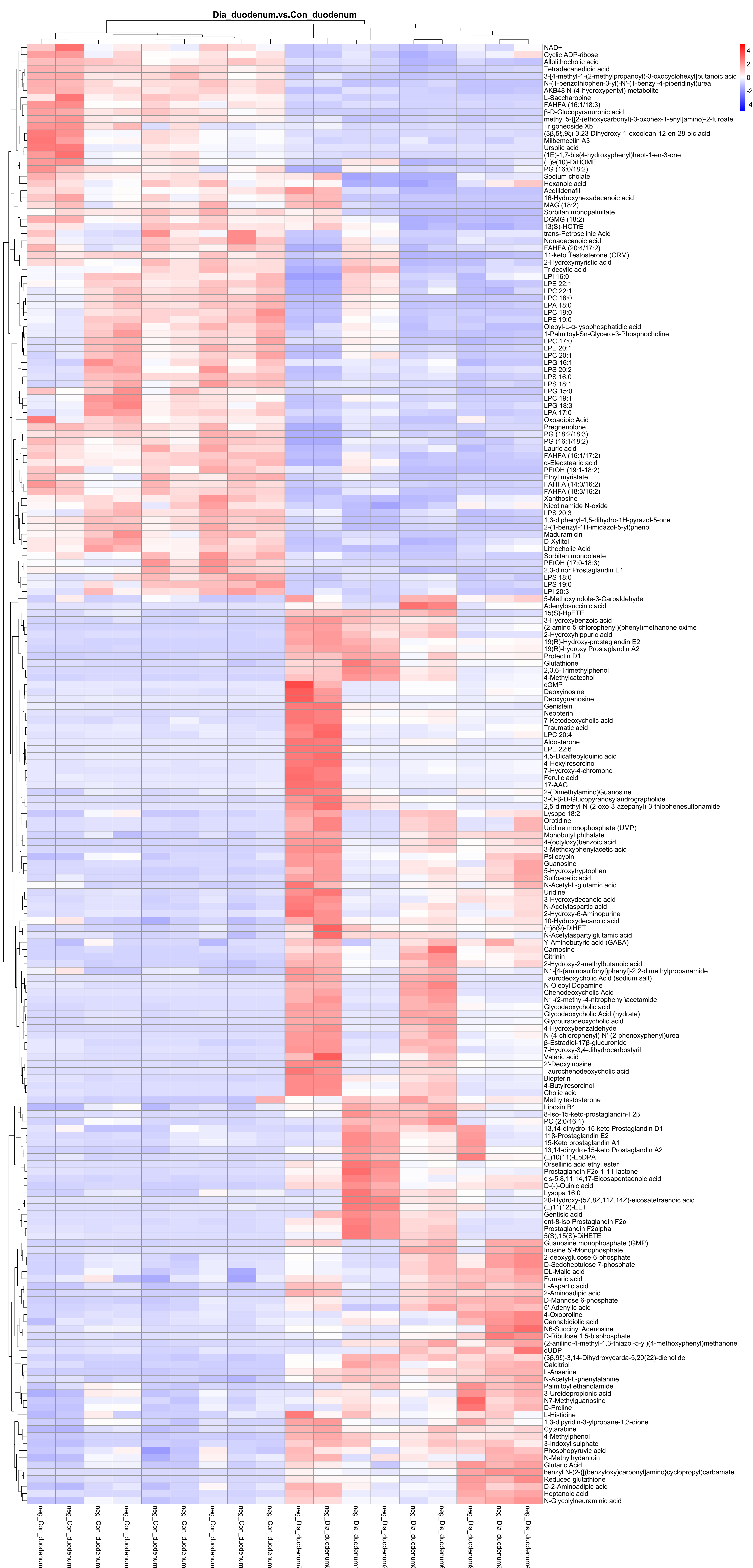

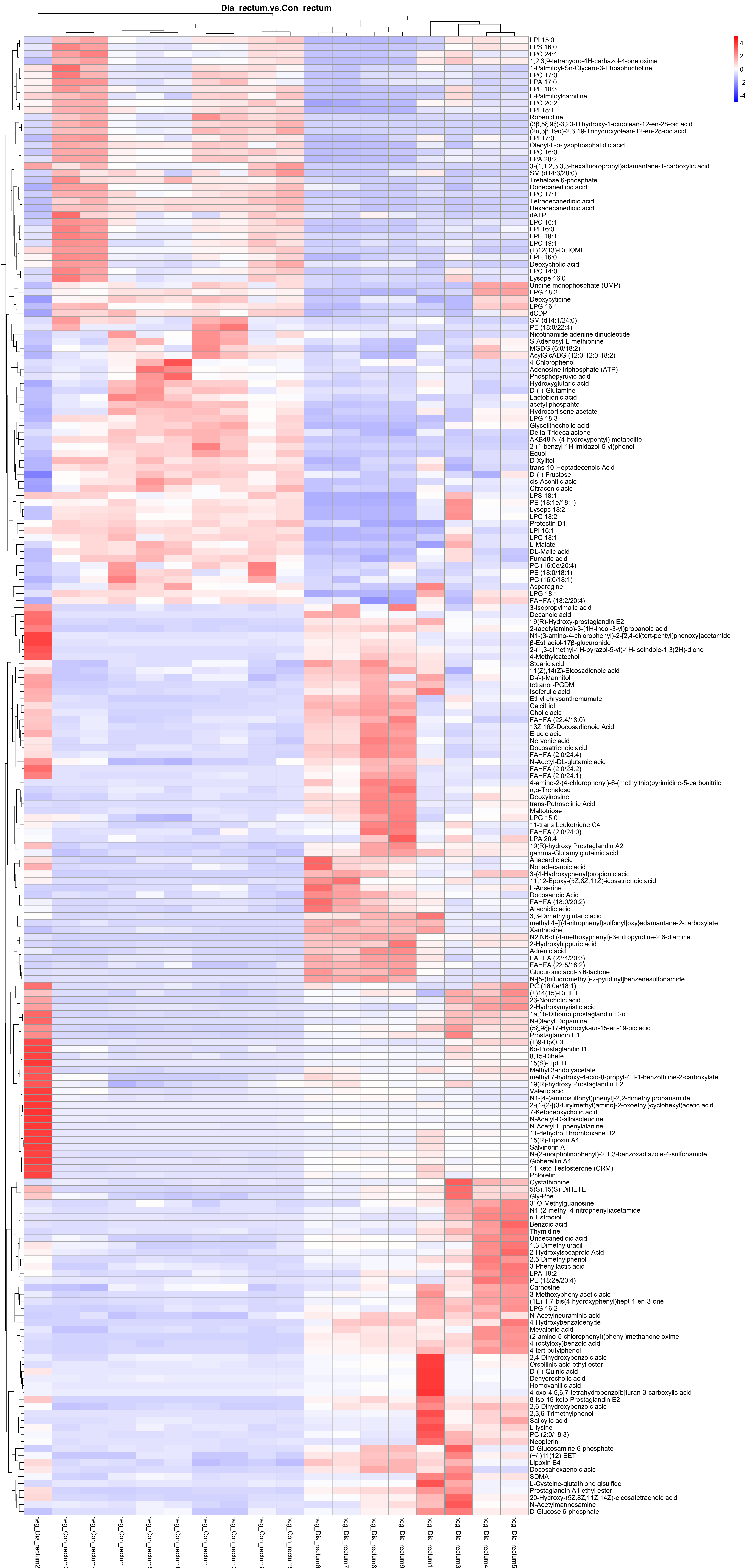

Supplement: Supplementary file 1 [file animals-11-01560-s001.zip › animals-1196821-supplementary-update/animals-1147480-supplementary/Supplemental Figure S3/Supplemental Figure S3-neg.pdf]

Dia\_colon.vs.Con\_colon

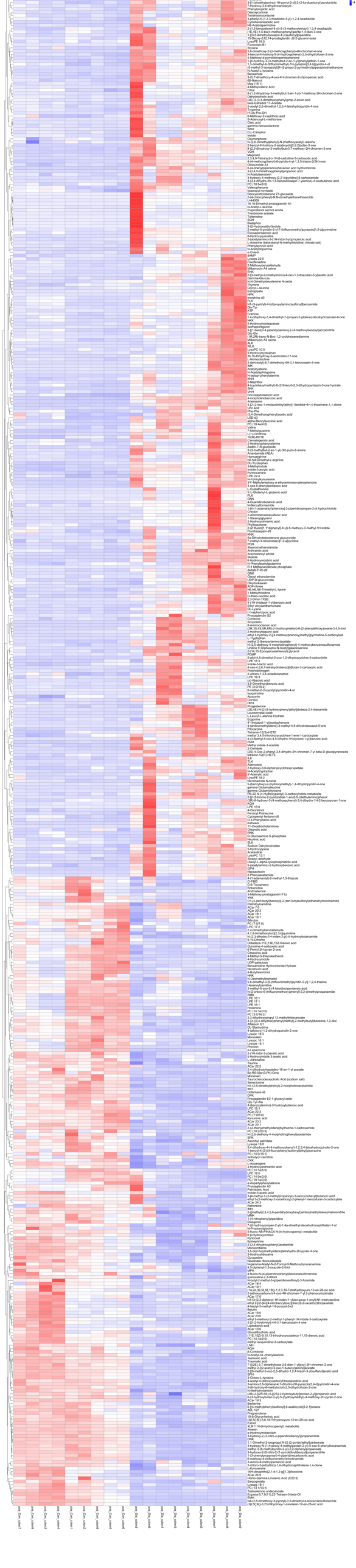



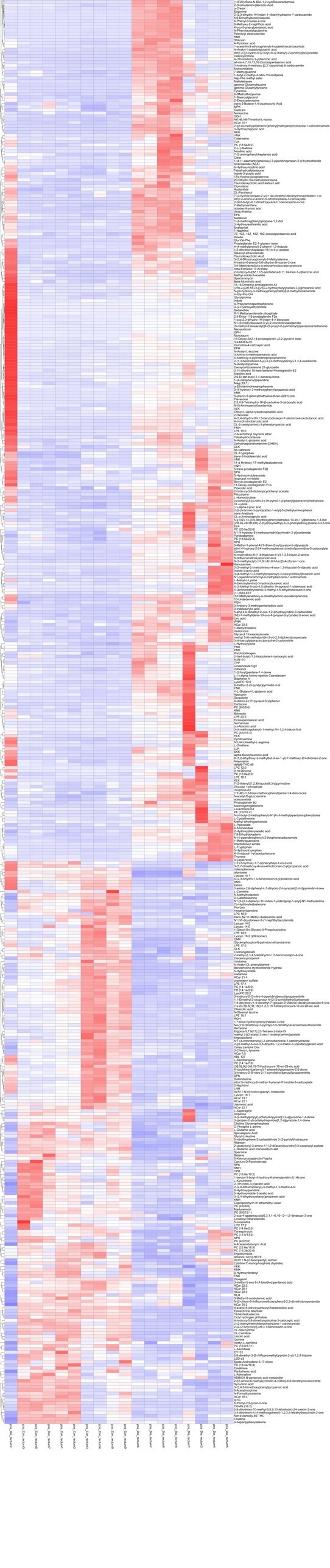

Supplement: Supplementary file 1 [file animals-11-01560-s001.zip › animals-1196821-supplementary-update/animals-1147480-supplementary/Supplemental Figure S3/Supplemental Figure S3-pos.pdf]

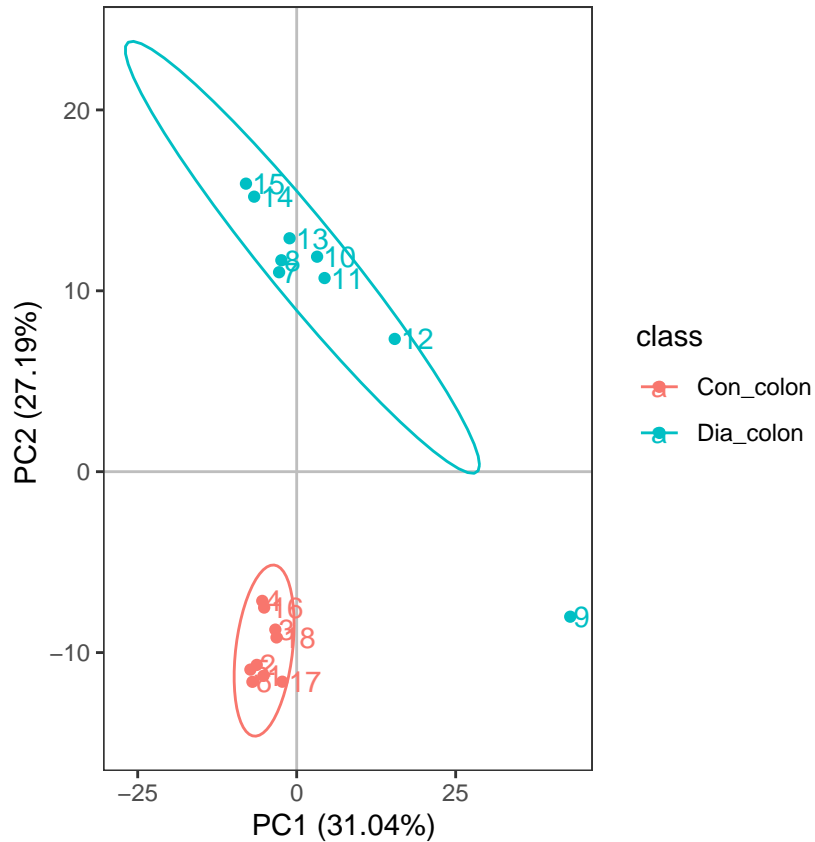

Supplement: Supplementary file 1 [file animals-11-01560-s001.zip › animals-1196821-supplementary-update/animals-1147480-supplementary/Supplementary Figure 2a PCA principal component analysis/colon_neg-PCA.pdf]

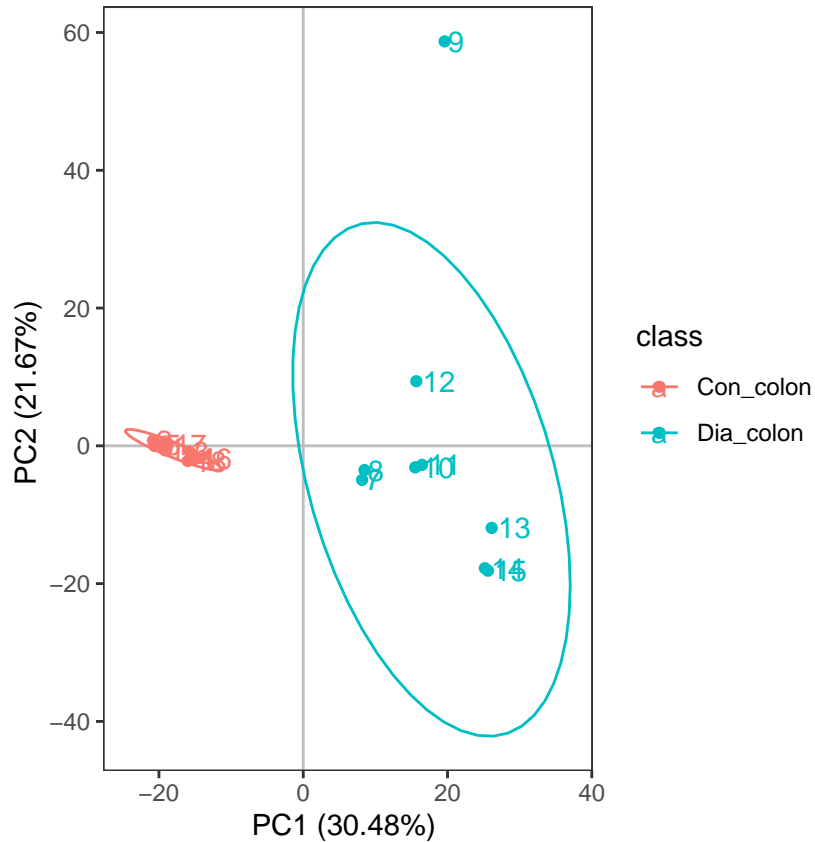

Supplement: Supplementary file 1 [file animals-11-01560-s001.zip › animals-1196821-supplementary-update/animals-1147480-supplementary/Supplementary Figure 2a PCA principal component analysis/colon_pos-PCA.pdf]

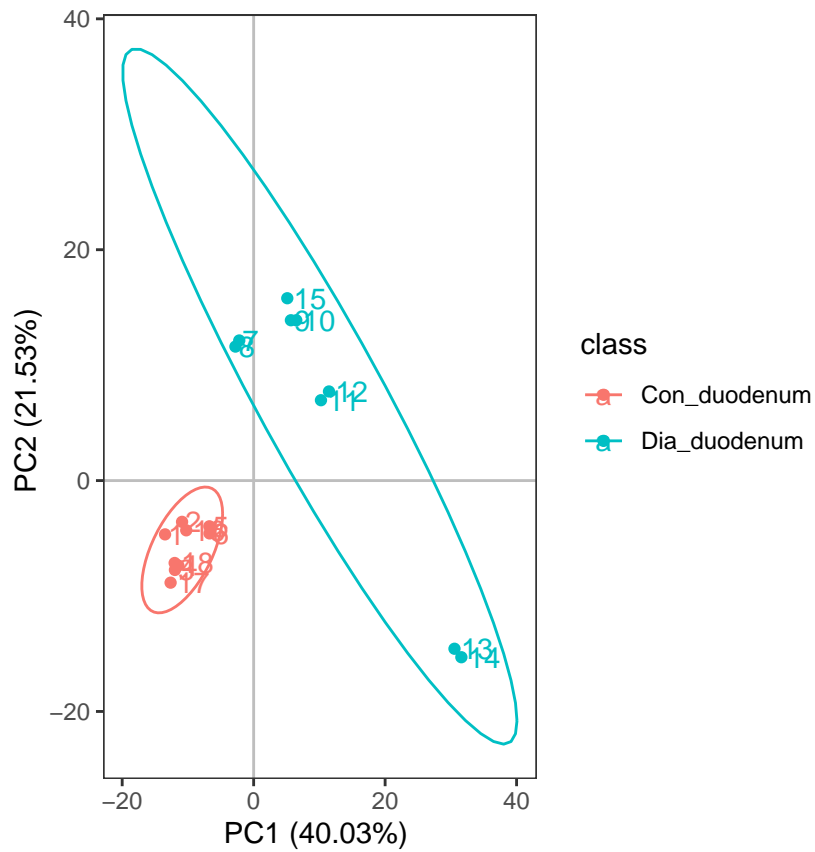

Supplement: Supplementary file 1 [file animals-11-01560-s001.zip › animals-1196821-supplementary-update/animals-1147480-supplementary/Supplementary Figure 2a PCA principal component analysis/duodenum_neg-PCA.pdf]

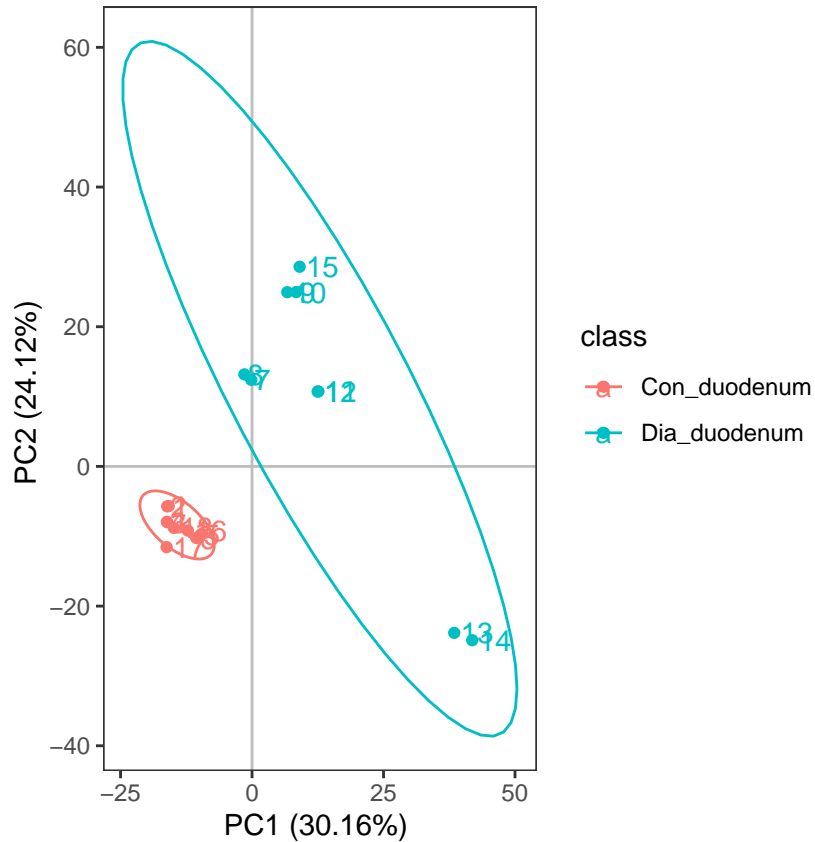

Supplement: Supplementary file 1 [file animals-11-01560-s001.zip › animals-1196821-supplementary-update/animals-1147480-supplementary/Supplementary Figure 2a PCA principal component analysis/duodenum_pos-PCA.pdf]

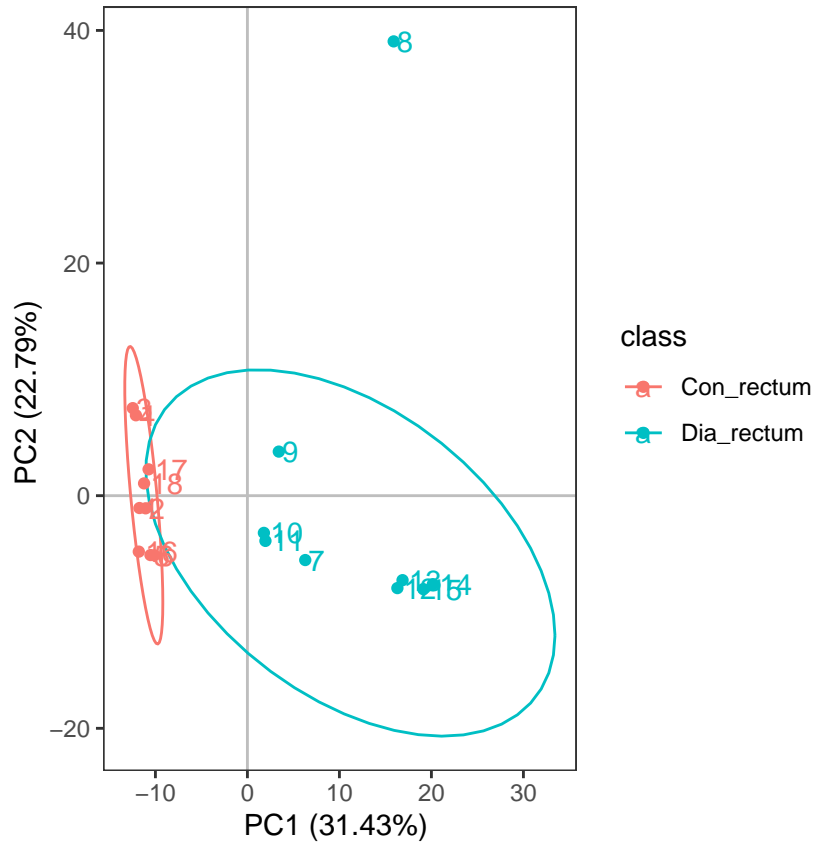

Supplement: Supplementary file 1 [file animals-11-01560-s001.zip › animals-1196821-supplementary-update/animals-1147480-supplementary/Supplementary Figure 2a PCA principal component analysis/rectum_neg-PCA.pdf]

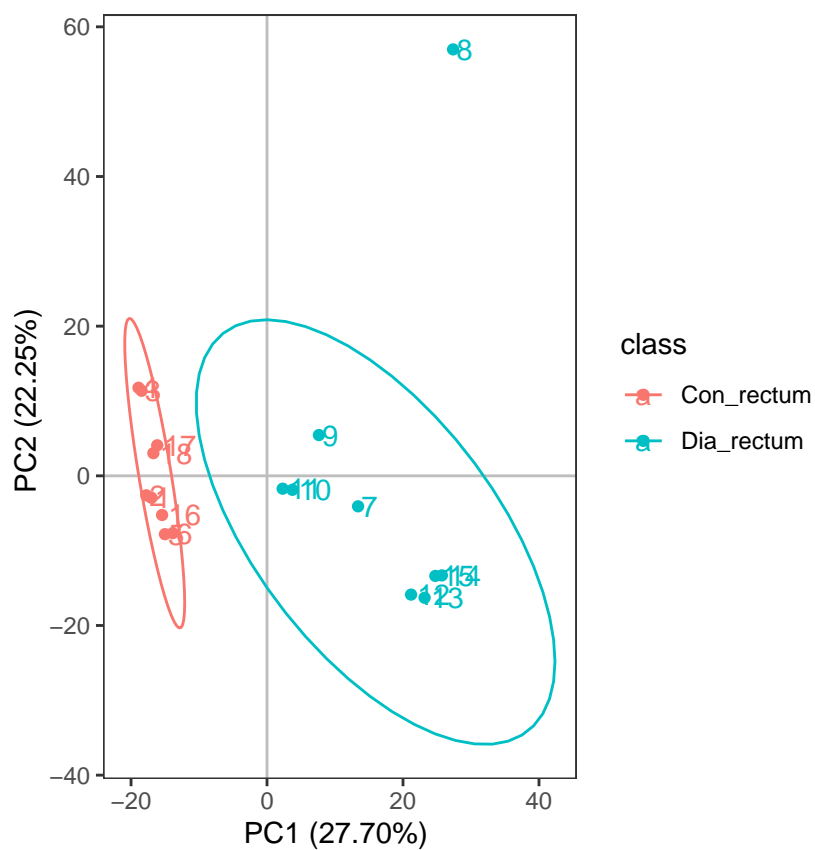

Supplement: Supplementary file 1 [file animals-11-01560-s001.zip › animals-1196821-supplementary-update/animals-1147480-supplementary/Supplementary Figure 2a PCA principal component analysis/rectumíñ_pos-PCA.pdf]

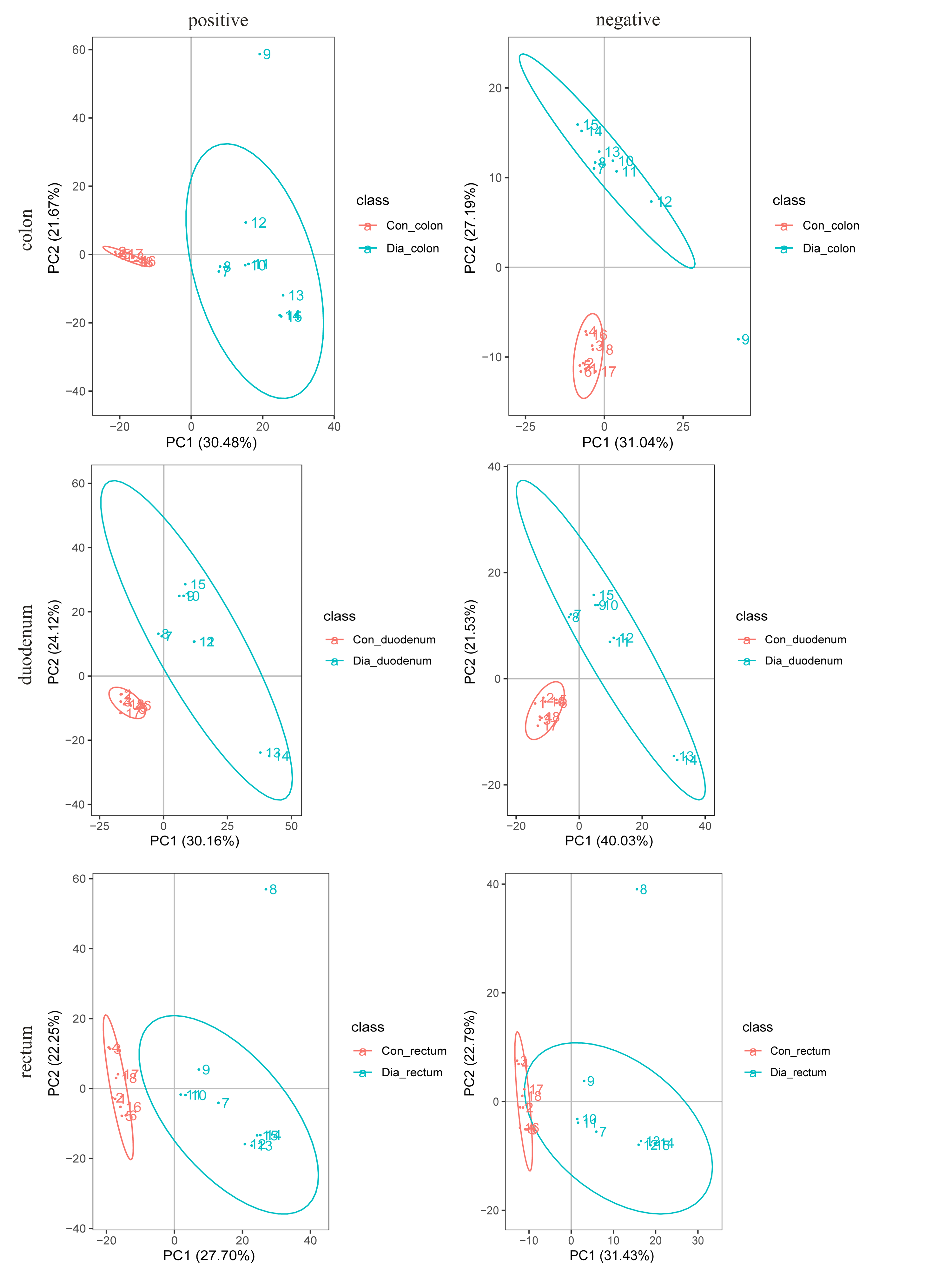

Supplement: Supplementary file 1 [file animals-11-01560-s001.zip › animals-1196821-supplementary-update/animals-1147480-supplementary/Supplementary Figure 2a PCA principal component analysis/Supplementary Figure 2a.tif]

class   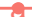 Con\_colon   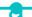 Dia\_colon

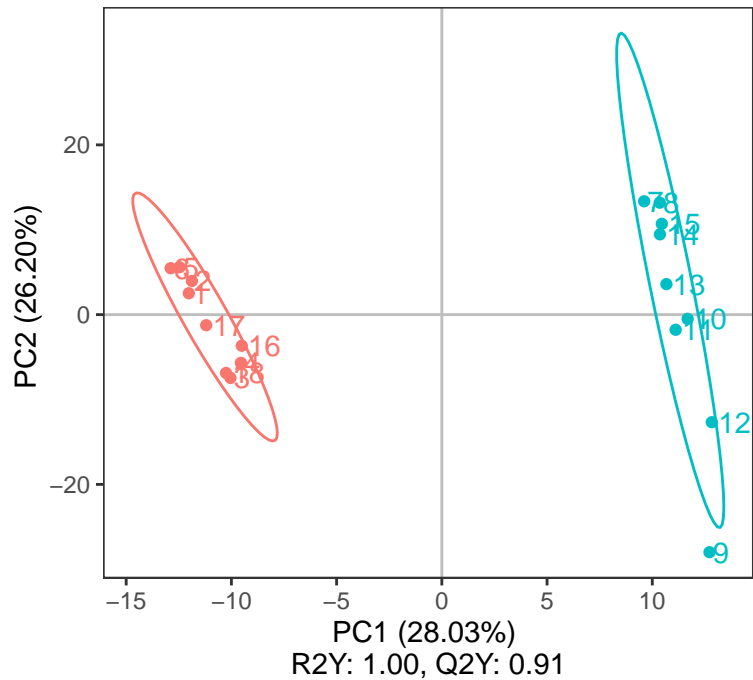

Supplement: Supplementary file 1 [file animals-11-01560-s001.zip › animals-1196821-supplementary-update/animals-1147480-supplementary/Supplementary Figure 2b PLS-DA scatter - point diagram and sort - verification diagram/Dia_colon.vs.Con_colon_neg_PLSDA-score.pdf]

Dia\_colon\_Con\_colon  
Intercepts:R2=(0.0,0.48), Q2=(0.0,-1.11)

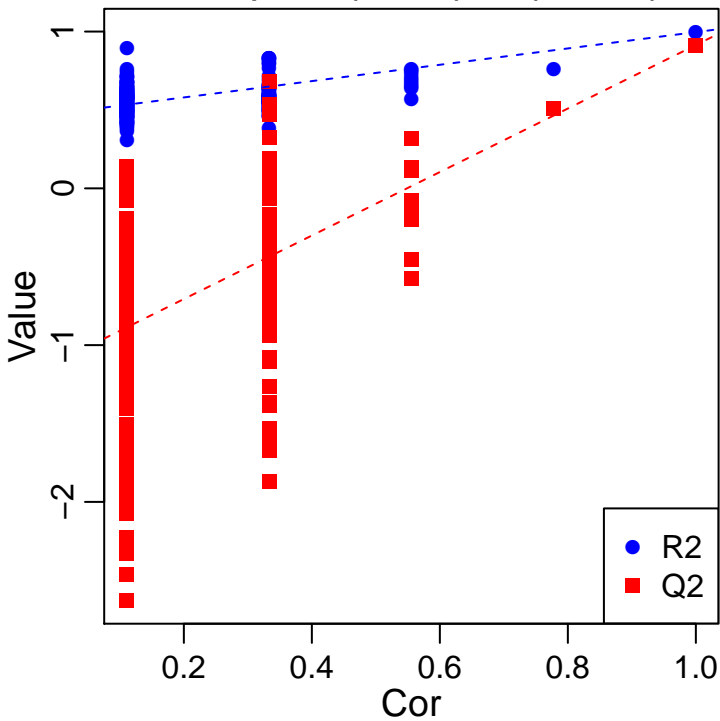

Supplement: Supplementary file 1 [file animals-11-01560-s001.zip › animals-1196821-supplementary-update/animals-1147480-supplementary/Supplementary Figure 2b PLS-DA scatter - point diagram and sort - verification diagram/Dia_colon.vs.Con_colon_neg_PLSDA-valid.pdf]

class   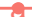 Con\_colon   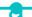 Dia\_colon

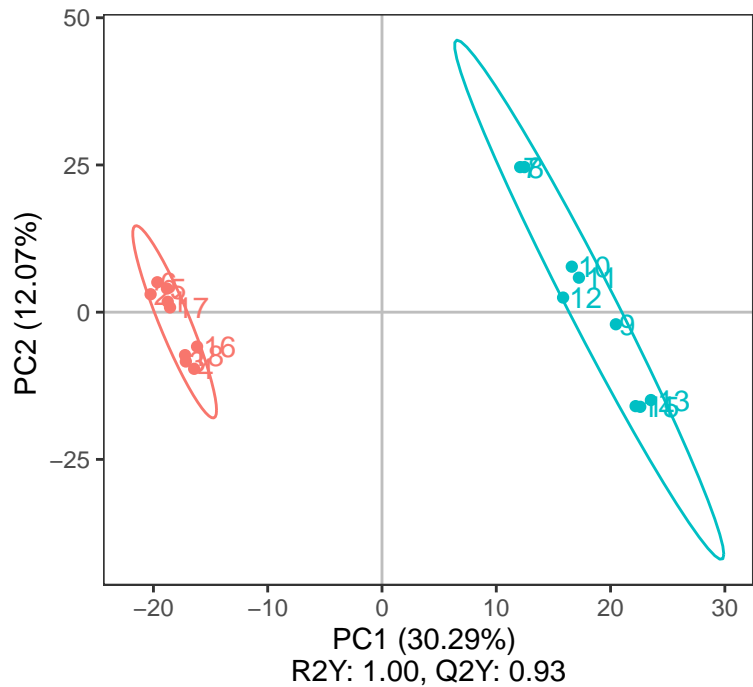

Supplement: Supplementary file 1 [file animals-11-01560-s001.zip › animals-1196821-supplementary-update/animals-1147480-supplementary/Supplementary Figure 2b PLS-DA scatter - point diagram and sort - verification diagram/Dia_colon.vs.Con_colon_pos_PLSDA-score.pdf]

Dia\_colon\_Con\_colon  
Intercepts:R2=(0.0,0.56), Q2=(0.0,-0.92)

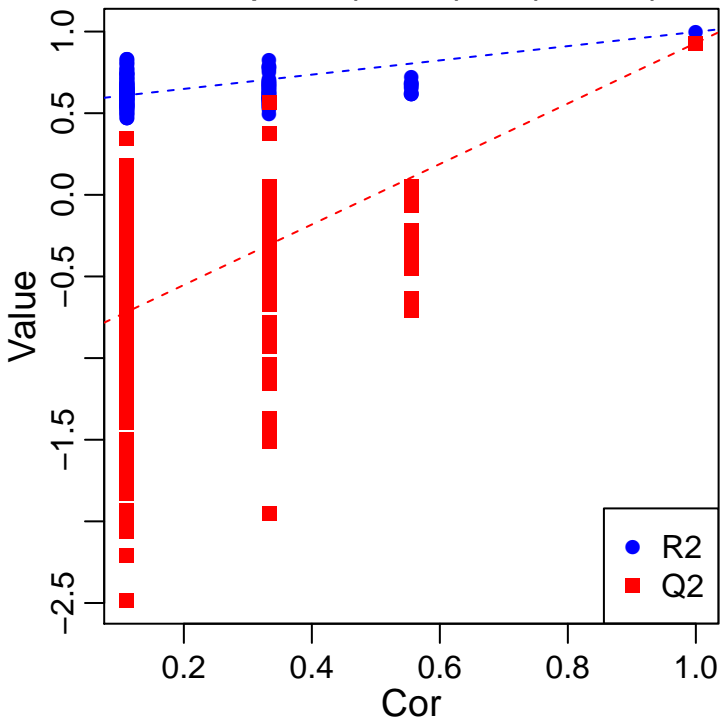

Supplement: Supplementary file 1 [file animals-11-01560-s001.zip › animals-1196821-supplementary-update/animals-1147480-supplementary/Supplementary Figure 2b PLS-DA scatter - point diagram and sort - verification diagram/Dia_colon.vs.Con_colon_pos_PLSDA-valid.pdf]

class    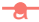 Con\_duodenum    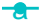 Dia\_duodenum

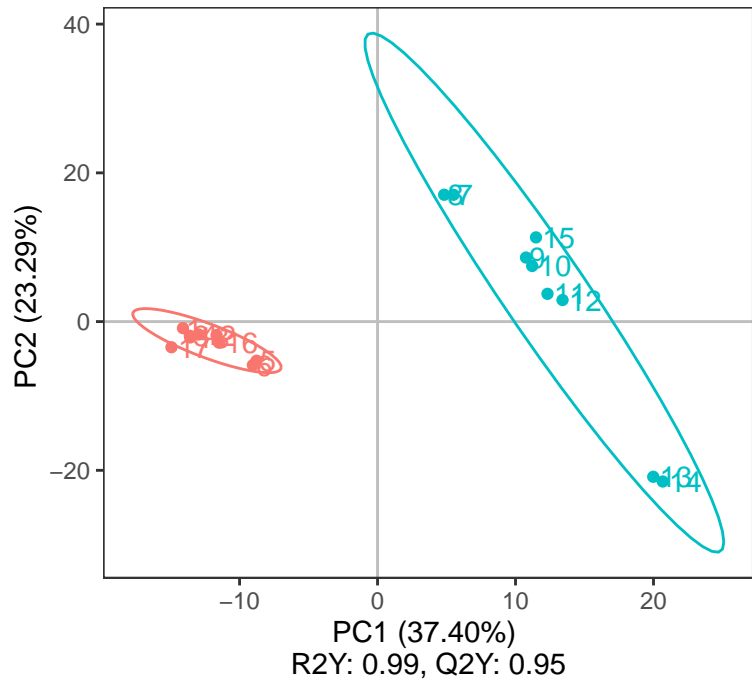

Supplement: Supplementary file 1 [file animals-11-01560-s001.zip › animals-1196821-supplementary-update/animals-1147480-supplementary/Supplementary Figure 2b PLS-DA scatter - point diagram and sort - verification diagram/Dia_duodenum.vs.Con_duodenum_neg_PLSDA-score.pdf]

Dia\_duodenum\_Con\_duodenum  
Intercepts:  $R^2=(0.0,0.39)$ ,  $Q^2=(0.0,-1.05)$

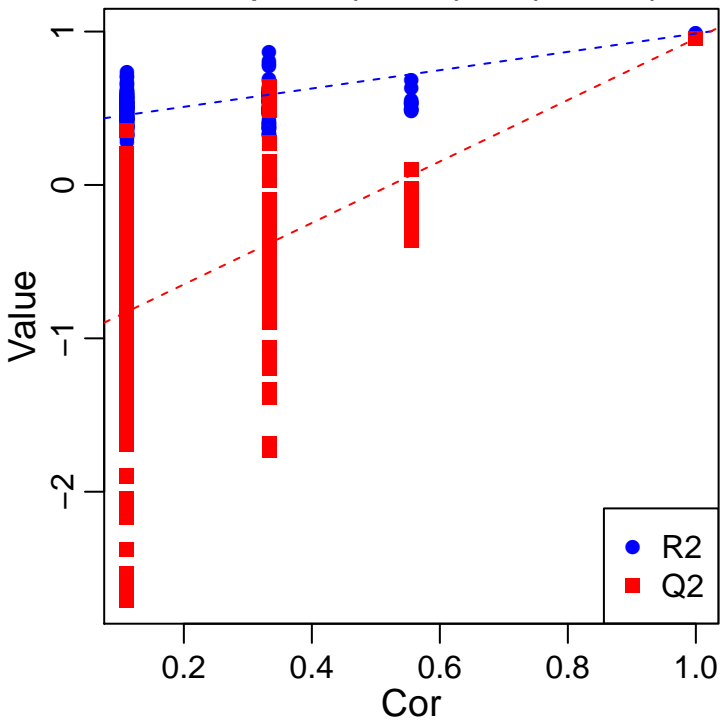

Supplement: Supplementary file 1 [file animals-11-01560-s001.zip › animals-1196821-supplementary-update/animals-1147480-supplementary/Supplementary Figure 2b PLS-DA scatter - point diagram and sort - verification diagram/Dia_duodenum.vs.Con_duodenum_neg_PLSDA-valid.pdf]

class    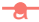 Con\_duodenum    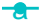 Dia\_duodenum

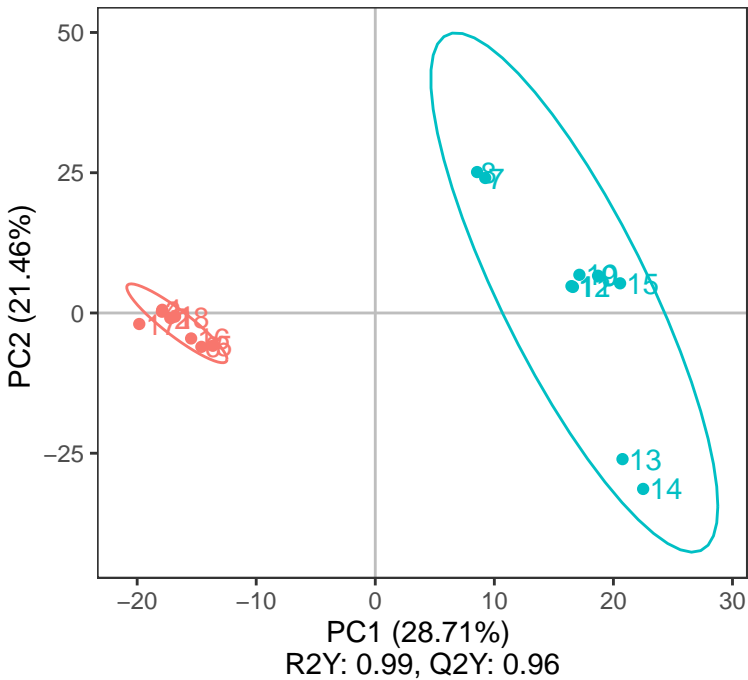

Supplement: Supplementary file 1 [file animals-11-01560-s001.zip › animals-1196821-supplementary-update/animals-1147480-supplementary/Supplementary Figure 2b PLS-DA scatter - point diagram and sort - verification diagram/Dia_duodenum.vs.Con_duodenum_pos_PLSDA-score.pdf]

Dia\_duodenum\_Con\_duodenum  
Intercepts:  $R^2=(0.0,0.56)$ ,  $Q^2=(0.0,-1.24)$

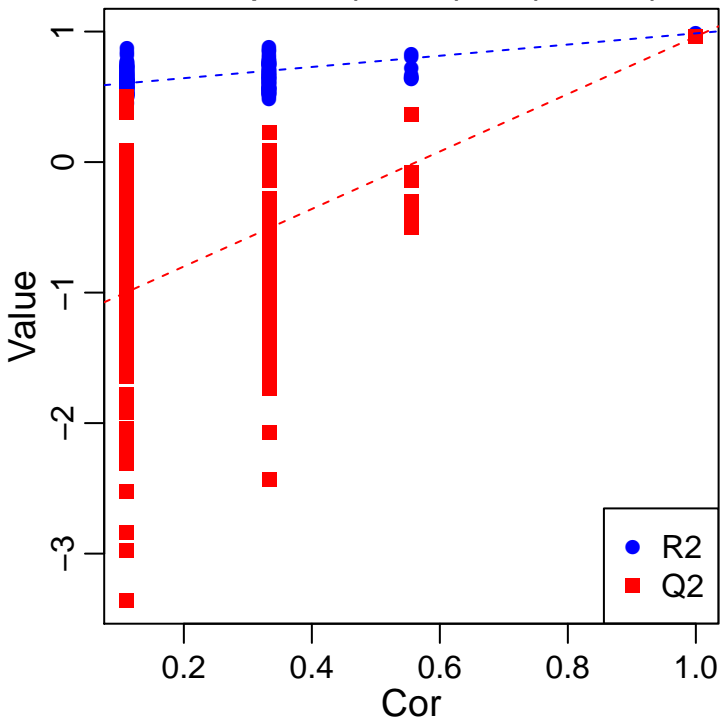

Supplement: Supplementary file 1 [file animals-11-01560-s001.zip › animals-1196821-supplementary-update/animals-1147480-supplementary/Supplementary Figure 2b PLS-DA scatter - point diagram and sort - verification diagram/Dia_duodenum.vs.Con_duodenum_pos_PLSDA-valid.pdf]

class    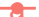 Con\_rectum    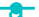 Dia\_rectum

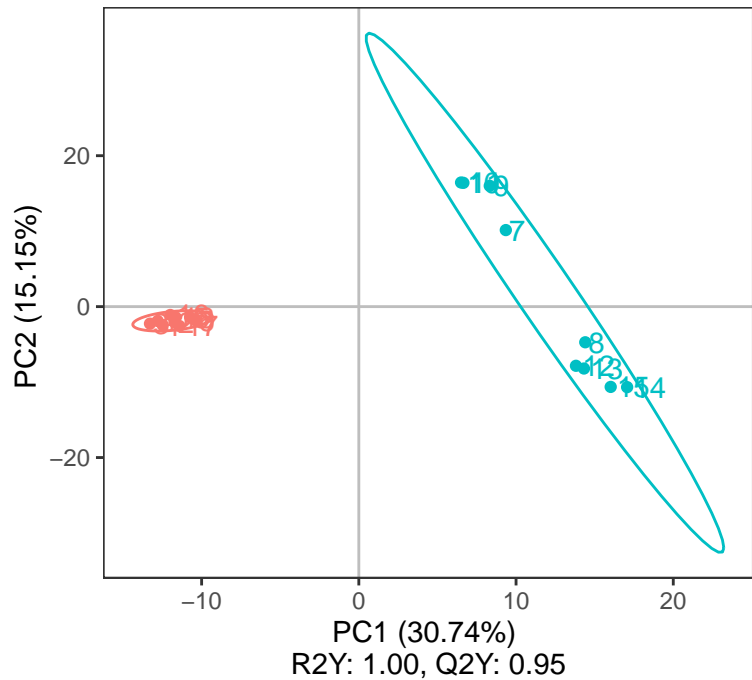

Supplement: Supplementary file 1 [file animals-11-01560-s001.zip › animals-1196821-supplementary-update/animals-1147480-supplementary/Supplementary Figure 2b PLS-DA scatter - point diagram and sort - verification diagram/Dia_rectum.vs.Con_rectum_neg_PLSDA-score.pdf]

Dia\_rectum\_Con\_rectum  
Intercepts: R2=(0.0,0.51), Q2=(0.0,-0.91)

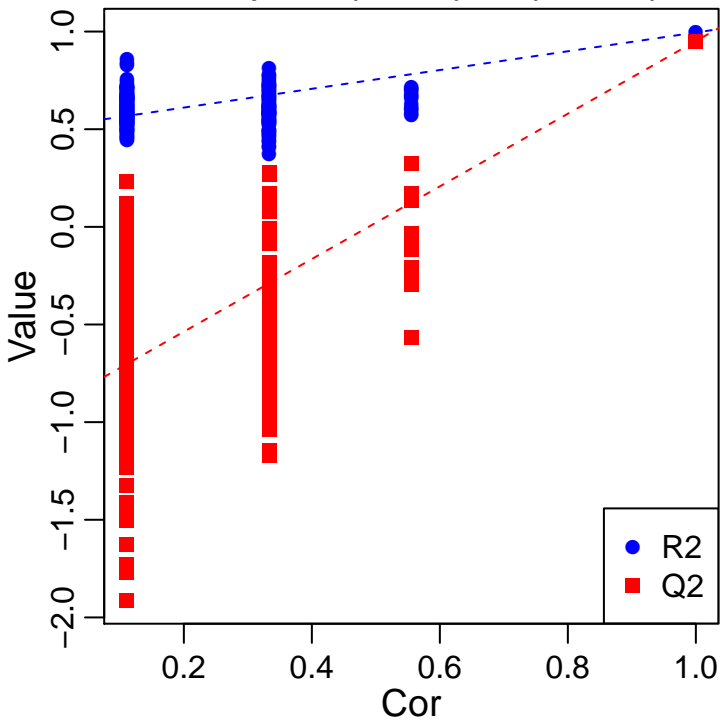

Supplement: Supplementary file 1 [file animals-11-01560-s001.zip › animals-1196821-supplementary-update/animals-1147480-supplementary/Supplementary Figure 2b PLS-DA scatter - point diagram and sort - verification diagram/Dia_rectum.vs.Con_rectum_neg_PLSDA-valid.pdf]

class    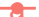 Con\_rectum    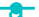 Dia\_rectum

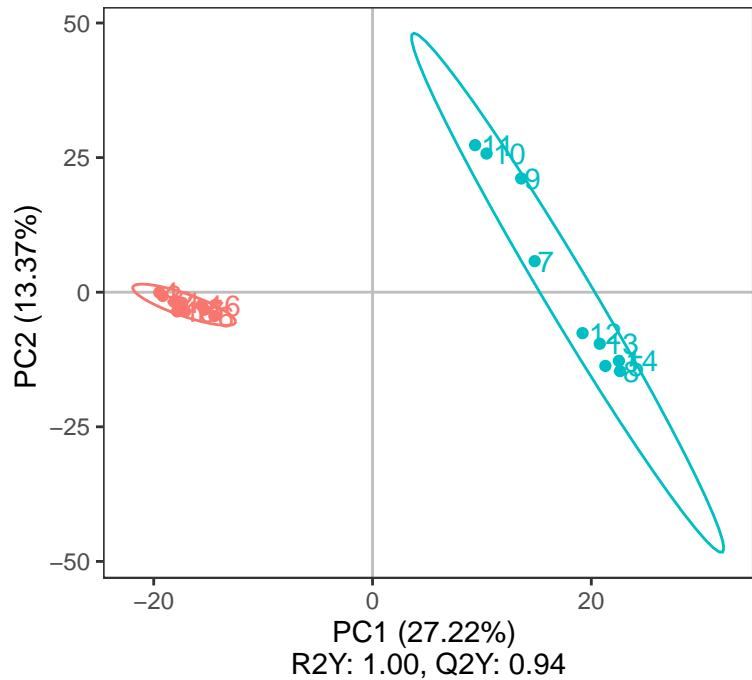

Supplement: Supplementary file 1 [file animals-11-01560-s001.zip › animals-1196821-supplementary-update/animals-1147480-supplementary/Supplementary Figure 2b PLS-DA scatter - point diagram and sort - verification diagram/Dia_rectum.vs.Con_rectum_pos_PLSDA-score.pdf]

Dia\_rectum\_Con\_rectum  
Intercepts: R2=(0.0,0.57), Q2=(0.0,-0.91)

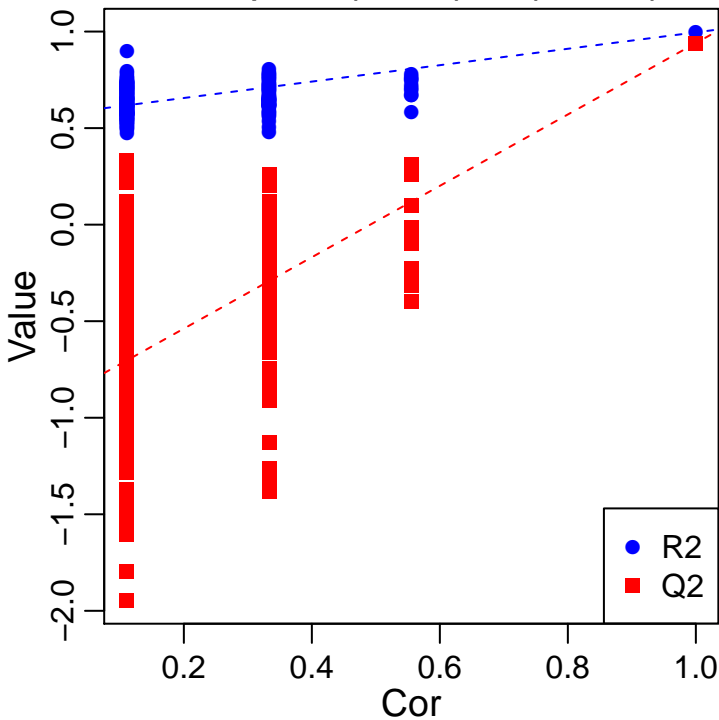

Supplement: Supplementary file 1 [file animals-11-01560-s001.zip › animals-1196821-supplementary-update/animals-1147480-supplementary/Supplementary Figure 2b PLS-DA scatter - point diagram and sort - verification diagram/Dia_rectum.vs.Con_rectum_pos_PLSDA-valid.pdf]

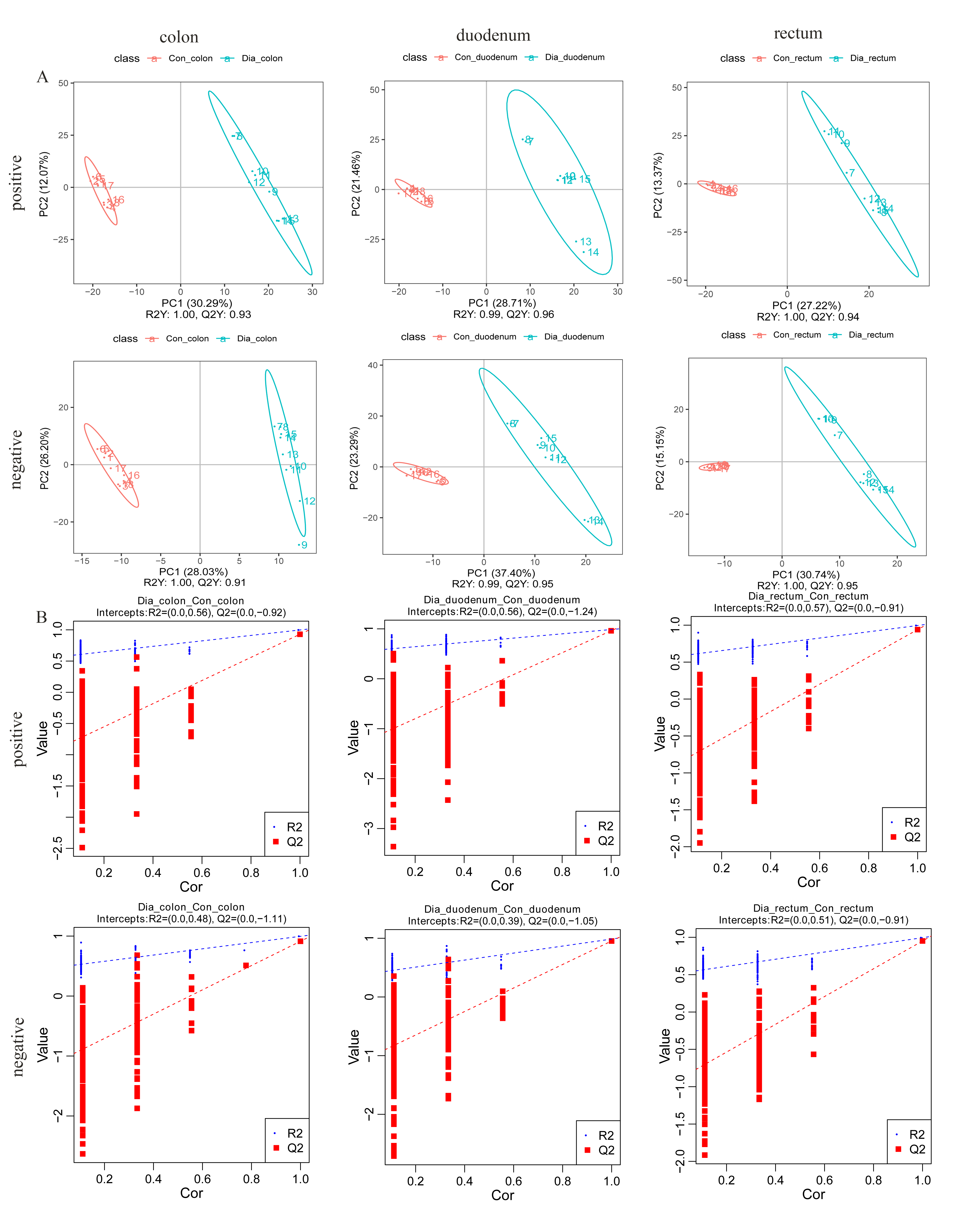

Supplement: Supplementary file 1 [file animals-11-01560-s001.zip › animals-1196821-supplementary-update/animals-1147480-supplementary/Supplementary Figure 2b PLS-DA scatter - point diagram and sort - verification diagram/Supplementary Figure 2b.tif]
